# Capacity Development of Local Self-Governments for Disaster Risk Management

**DOI:** 10.3390/ijerph181910406

**Published:** 2021-10-02

**Authors:** Vladimir M. Cvetković, Jasmina Tanasić, Adem Ocal, Želimir Kešetović, Neda Nikolić, Aleksandar Dragašević

**Affiliations:** 1 Faculty of Security Studies, University of Belgrade, Gospodara Vučića 50, 11040 Belgrade, Serbia; zelimir.kesetovic@fb.bg.ac.rs; 2 Scientific-Professional Society for Disaster Risk Management, Dimitrija Tucovića 121, 11040 Belgrade, Serbia; 3 Standing Conference of Towns and Municipalities, 11103 Belgrade, Serbia; jasmina.tanasic@skgo.org; 4 Independent Researcher, Ankara 06500, Turkey; ocadem@gmail.com; 5 Faculty of Technical Sciences, University of Kragujevac, 32102 Čačak, Serbia; neda.nikolic@ftn.kg.ac.rs (N.N.); aleksandar.dragasevic@ftn.kg.ac.rs (A.D.)

**Keywords:** disaster, risk management, capacity development, local self-governments, Serbia

## Abstract

The objective of this research was to examine the capacity development of local self-governments in the field of disaster risk management (DRM). This quantitative research examines the degree of implementation of strategic, legislative, and institutional frameworks, as well as the capacity of local authorities to apply related policies through five analytical scopes: (1) degree of preparedness and legal framework; (2) financial framework; (3) policy aspects; (4) cooperation and partnership; (5) communication. The ability of municipalities and towns to respond to disasters was also analyzed and compared. In this paper, our initial hypothesis was that the effective implementation of the concept of DRM policy in towns of Serbia requires the continual strategic, tactical, and operational transformation of the public administration and public management system in order to strengthen the capacity of local self-governments for disaster prevention, preparation, response, and recovery. This multimethod research was conducted over the period of 2014–2017 and included the following two target groups: (a) heads of disaster sectors in local self-government units (mayor) and (b) employees of the DRM sector in local self-government units. The results of this research will enable decision makers to successfully respond to challenges and help to improve the capacity of local self-governments and public local administrations within the scope of DRM in the Republic of Serbia, based on the principles of prevention and proactive action, coordination, cooperation, partnership, and responsibility.

## 1. Introduction

The disaster management process is defined as all stages of intervention, recovery, reconstruction, harm reduction, and disaster preparedness that occur after a disaster event and continue until the next disaster occurs [1]. Disaster management activities must be considered at all stages of a country’s development. In this context, it is preferable to include all groups of society at all phases of governance, reducing the risk of disasters and achieving a sustainable development trend. For this purpose, educational programs aimed at ensuring that people who make up society acquire the necessary knowledge and skills to reduce the possible damage from disasters are put on and participation in these programs is ensured at the highest level [2].

One of the most important activities for reducing disaster losses is capacity building. Considering that a disaster occurs when a system or community’s capacity and response capabilities are exceeded [3,4,5,6], it is vital to be aware of including capacity-building work early in the disaster management process. There is no universally accepted definition of capacity building [7,8,9,10]. Since the mid-1990s, capacity building has been a focal point of disaster management thinking and practice. This term refers to the process of strengthening the ability of individuals, organizations, or systems to perform effective and long-term functions [11]. As a result, capacity building describes an increase in the ability of interested individuals or groups to adapt to unusual events in their own lives [12]. Insufficiently developed capacities unequivocally cause disaster management problems, which are defined in the literature as a pattern of inconsistent and often weak performance by local governments across all disaster stages resulting from a lack of adequate resources and legitimacy for the disaster planning process [13].

Capacity building in disaster management [14] seeks to create human resources that can act together to deal with hazard events as well as to supply the necessary equipment and make appropriate administrative arrangements [15]. More commonly, corporate capacity building can be expanded through structural transformation activities, disaster management training, support programs, and corporate human resource development in collaboration with financial and technological resources [16]. Furthermore, capacity building aims to achieve measurable and sustainable results through cooperation between individuals, groups, networks, or communities through scenario-based processes [17,18].

Capacity-building plans should be integrated into the disaster management system, covering international, country, region, province, and district (town) planning processes, as well as improving quality of life by increasing both individual and societal coping capacities during the risk reduction phase [19]. Government and civic organizations (NGOs, Red Cross, etc.) should work decisively together to build the community’s disaster-response capacity. There are numerous publications [8,20,21,22,23] that discuss the importance and applications of capacity building; however, the academic dimension of capacity building for disaster mitigation has not been sufficiently explored [24].

Serbia’s land is sensitive to a variety of natural hazards. The risk is not uniform across the country and varies based on the kind of hazard involved and the estimated damage potential [25]. Seismic hazards, landslides, rock falls, floods, torrential floods, excessive erosion, droughts, and forest fires are some of the significant natural hazards that could occur within the territory of Serbia [26]. The current state of protection against natural disasters in the territory of Serbia is characterized by the incompleteness and unavailability of information on the risks of possible natural disasters and on the consequences they may have, as well as insufficient “public participation”. The insufficient capacity of local authorities, professional services, and consultants to engage in a modern approach to DRM is evident [27]. The current situation is also characterized by the lack of a single database on the spatial distribution of certain natural disasters—i.e., the determination of potentially critical zones (floods, landslide cadasters, torrents, etc.). The condition of the overall system of protection against natural disasters in the territory of Serbia is not satisfactory, especially in relation to the spatial aspects of DRM [28].

From the end of the 1990s until 2009, in the Republic of Serbia the disaster response system lacked a single legislative, strategic, and institutional framework. Responsibilities for responding to disasters were divided between different departments: the Ministry of Interior; the Ministry of Defense; the Ministry of Agriculture, Forestry, and Water Management; the Ministry of Health; and the Ministry of Environment and Physical Planning. In March 2009, Conclusion 05 No. 02-1312/2009 was adopted by the government of the Republic of Serbia, which established a working group composed of representatives of the above-mentioned ministries. The task of the working group was to analyze the current situation in the field of emergency situations, propose the harmonization of existing legal norms, and adopt new regulations, with the aim of unifying competencies in this area. The Law on Emergency Situations was adopted in 2009 and remained in force from 2010 until 2018, when it was brought under the law on disaster risk reduction and emergency management [29]. This law, among other things, establishes the obligation to perform threat assessments and create protection and rescue plans at all levels. The response to a crisis is determined according to the bottom-up system—that is, by the local self-government and local community where the crisis began.

After the May floods, in July 2014 [30] the Law on Elimination of the Consequences of Floods in the Republic of Serbia [31] was passed, and the Office for Relief and Reconstruction of Flooded Areas was established by decree of the President in 2015; this grew into the Office for Public Investment Management, which is specified by the law on reconstruction after natural and other disasters. The National Strategy for Protection and Rescue in Emergency Situations [32] identified four groups of shortcomings in the existing protection and rescue system: institutional-organizational; material and technical; shortcomings in the cooperation, coordination, and availability of information; the lack of human resources and education. The institutional and organizational shortcomings of the existing system relate, among other things, to the lack of conditions for the consistent application of regulations, the non-implementation of preventive measures; the uneven distribution of service capacities across the territory of Serbia, and the nonestablishment of system 112. Insufficient cooperation and coordination at both the horizontal and vertical levels, as well as the need to improve international cooperation, were highlighted as a separate group of problems. Lastly, shortcomings related to unpreparedness, the low level of local self-government capacity, and the underdeveloped culture of prevention were mentioned.

For these reasons, strengthening the DRM system through research, development, and the implementation of innovative solutions in this area can reduce disaster risk, which will directly affect the level of safety of citizens and their resilience to the consequences of disasters. In addition, the implications will lead to improving the security culture and resilience of individuals and the community to the consequences of disasters. As a result, enhancing the disaster risk management system via research, development, and the implementation of new solutions in this field can minimize disaster risk, directly affecting the public’s safety and resistance to the effects of disasters.

**Figure 1:**
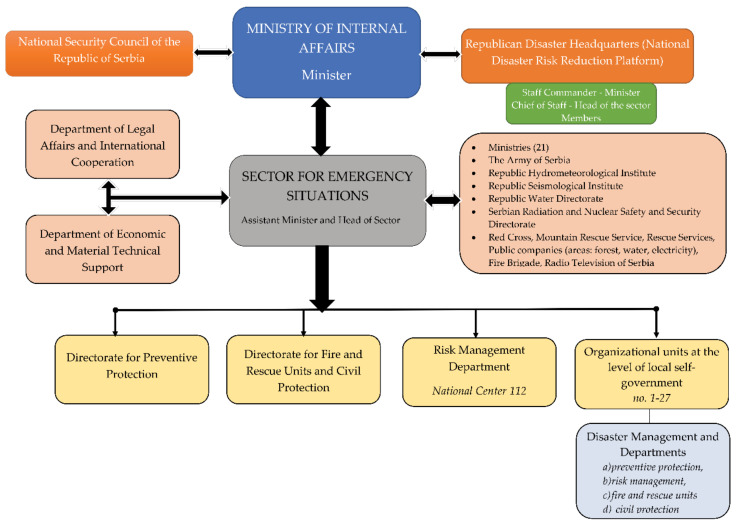
Serbia’s disaster risk management systems.

Disaster management in Serbia, in general, has bureaucratic characteristics (Figure 1): there are many actors, none of whom has too much power; decisions are compromised; and their implementation is ineffective [33]. Decision-makers’ perceptions and experiences, cultural patterns, and the ways of setting priorities among political elites all play a role in timely observation and disaster preparedness [34]. That is why, at all stages, the concepts of organizational structure and organizational cultures are useful for understanding organizational disaster management and disaster management policy.

### Literature Review

Building social capacity to reduce disaster risks will be beneficial for human welfare. It is not sufficient to improve the capacity of the central government alone to reduce disaster-related damage. Local governments, provinces, districts, and towns will also be more effective in responding to disasters in a timely and effective manner if their disaster management capacities are strengthened. This approach will result in the better coordination of national and international actors and the central government during disasters, as well as in the more efficient use of resources. Capacity building is typically handled at three levels: individual, institutional, and systemic [35].

Individual capacity building is essential because it improves knowledge, skills, values, attitudes, health outcomes, awareness, and motivation. It requires the creation of conditions that encourage participation. Organizational processes such as organizational culture and leadership are referred to by terms such as capacity development, human resources, physical resources, various networks, punishment and reward systems, and performance. Organizational capacity building determines how an individual can contribute to an organization. In an organizational-based approach, the community is at the center of disaster risk management and all stages of this approach (diagnostics, analysis, improvement, monitoring, and evaluation) are organized around the community’s interests and capabilities. In this approach, all activities are carried out by members of the community [35] and society must be structured to raise awareness of the need for disaster management. The environment and conditions required for developing the capacities of individuals and organizations are linked to corporate capacity building. According to these definitions, capacity building can be defined as the development of skills to reduce and cope with disasters and disaster risks in communities at the local, provincial, and national levels [36].

The need for flexibility in disaster risk reduction efforts constrains the implementation of appropriate capacity building assessment studies for disaster risk reduction [37]. Terminologically, ownership in the local context, capacity assessment, roles and responsibilities, the diversity of activities and methods, monitoring, evaluation, and learning are all important components of DRR capacity building [8]. Additionally, some studies show that political leaders lack adequate DRR training [38] and the analysis of data from a huge number of qualitative interviews indicates that there are discrepancies between theory and practice in disaster risk reduction capacity building [8]. Additionally, it was found that achieving policy changes for DRM requires certain conditions, such as the perception of a problem in need of a solution and the perception that legal and hierarchical accountability need to be improved [39].

Capacity building has largely been reported in Sendai in order to mitigate disaster risks [40] and has been identified as a means to substantially reduce global disaster losses [8]. Furthermore, community empowerment for DRM requires their engagement in risk assessment, mitigation planning, capacity building, implementation, and the creation of monitoring systems, all of which assure their stake in the outcome [41]. The preparatory stage in the predisaster period refers to the activities carried out prior to a disaster. At this stage, it is critical to minimize damage and take the necessary technical, managerial, and legal measures to ensure that society can get deal with dangerous situations in a way that creates minimal damage. It is critical to highlight the two main stages prior to a disaster: damage reduction and pre-preparation. Forecasting (if possible), giving early warnings, and disaster impact analyses are all carried out during the preparation phase. Differences in community resources, livelihood alternatives, and assets have an impact on local capacity and the degree to which it may be enhanced [42,43].

It has been stated that there are systematic issues between the theoretical principles and current performance of capacity building in DRR. The main issue is the variable and unpredictable nature of disasters. Cognitive tendencies and formal approaches to combating this problem may have consequences that exacerbate rather than solve the problem [44]. Additionally, it has been stated that changes at the local level should only be encouraged in a weak way [45].

Capacity building for disaster mitigation is the process of developing skills to ensure that disasters have the least impact on individuals, institutions, and society [46]. In capacity building, the type of disaster involved must be addressed in a social and political context and planned for at the local level; individuals and communities on a local scale must be prioritized in disaster mitigation capacity building [47]. However, local ownership is critical to the success of such projects [48]. In previous studies, the need for capacity building to reduce disaster risks to local people exposed to disasters has been highlighted [49]. One of the key concepts in developing capacity to reduce long-term disaster losses is sustainability. The concept of sustainability in disaster loss reduction is a driving force of disaster risk reduction in the long term which means that society must be constantly prepared and aware of the reduction in potential disaster losses [50].

The objective of this research is to examine the capacity development of local self-governments in the field of DRM. In a broader sense, this study analyzes the strategic, legislative, and institutional frameworks, as well as the capacities of local authorities to apply disaster risk management policies through the following four analytical aspects:

- Degree of preparedness and legal framework of DRM;
- Financial framework of DRM;
- Policy aspects (strategic and operational levels of DRM);
- Cooperation and partnership (relations between the state, local authorities, and citizens within the DRM concept; formal obligations of the citizens in disaster circumstances as well as their expectations of the state; the engagement of citizens in disaster management; the role of civil society and citizen organizations; regional and international cooperation);
- Communication (DRM communication, manner of providing information about disaster events, establishing communication channels, assigning liability within the concept of disaster risk communication, the monitoring and evaluation of DRM, building safety culture, the education of local self-governments in the realm of disaster risk management, providing education to citizens and encouraging their active participation, creating databases and websites that are continuously updated with disaster-related data).

## 2. Materials and Methods

This paper focuses on the general hypothesis that the effective implementation of DRM policy in the towns of Serbia requires the continuous strategic, tactical, and operational transformation of the public administration and public management systems so as to enable local self-governments to prevent disasters from occurring and prepare communities to respond to and recover from possible disaster events. The capacities of municipalities and towns to respond to disasters were also analyzed and compared.

This research was conducted in the period 2014–2017 and focused on the following two target groups: (a) heads of disaster sectors (mayors) in local self-government units (mayor) and (b) employees of the disaster risk management sector in local self-government units (105 local self-governments).

The first part of this research focused on mayors, who were asked to fill out a questionnaire made up of eight questions relating to the four surveyed dimensions of DRM. The questionnaire was in the form of a Likert scale of attitudes. The questionnaire was answered by 17 out of 23 mayors in the period July–September 2017 (Appendix A). The research was conducted in the following 23 towns: Belgrade, Valjevo, Vranje, Zaječar, Zrenjanin, Jagodina, Kragujevac, Kraljevo, Kruševac, Leskovac, Loznica, Niš, Novi Pazar, Novi Sad, Pančevo, Požarevac, Smederevo, Sombor, Sremska Mitrovica, Subotica, Užice, Čacak, and Šabac.

In the second part of this research, the attitudes of employees of the DRM sector in local self-government units were examined. In this, 105 local governments in the Republic of Serbia were included (Supplementary Materials). The aim of this research, or its social and practical objective, was to improve the policy framework of DRM at both the local and national levels, as well as increasing the capacities of local administrations and other related institutions and organizations in the local community within the scope of risk management.

### 2.1. Study Area

**Figure 2:**
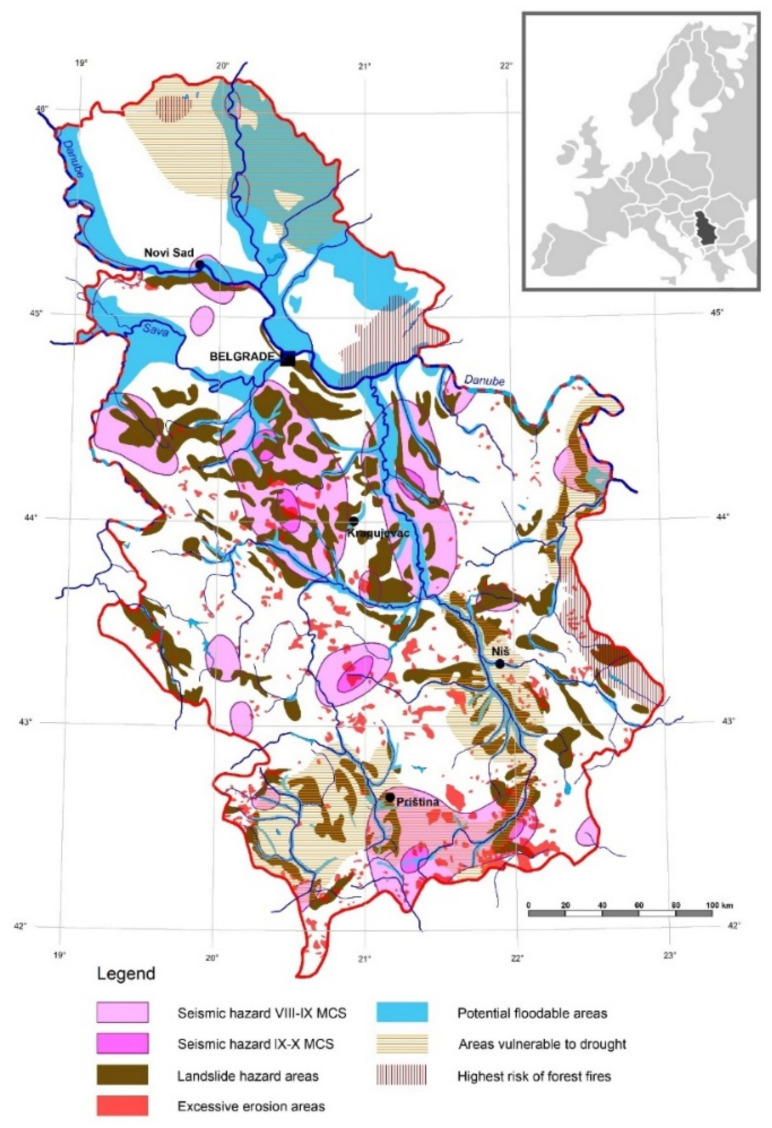
Integral vulnerability map of the natural hazards in the territory of Serbia [25].

Covering an area of 88,499 km^2^, the Republic of Serbia is located at the crossroads of Central and Southeastern Europe in the Southern Pannonian Plain and the central Balkans (Figure 2). In order to better understand the socioeconomic context of the local self-governments included in the survey, an overview of common indicators is given in this paper. The geographical, demographic, and socioeconomic factors of cities are important because they provide a framework for DRM. The estimated number of inhabitants in cities, the area they occupy, their population density, the number of settlements they contain, the number of unemployed persons registered with the national employment service, the number of beneficiaries of social financial assistance, and the expected duration of live births are indicators used to describe the socioeconomic context. These are important because they form the basis for planning and implementing responsibilities in the field of DRM. Out of the total number of local self-government units, about 39% of the total population of Serbia live in 23 cities. Cities differ a lot in terms of their population, the area they occupy, their population density, and their number of settlements (Appendix B).

The factors of population, area, population density, and number of settlements vary greatly among the cities of Serbia [51]. Due to its size and specificities, Belgrade stands out among all the cities of Serbia. With the largest population and the greatest area occupied (the total area covers 322,268 ha), Belgrade occupies the most prominent position. With its 17 municipalities, it is an entity characterized by all forms of diversity. As for the population of other cities of Serbia, Novi Sad, Nis, and Kragujevac follow on from Belgrade, whereas Pirot, Zaječar, and Kikinda have the lowest population. Additionally, the most densely populated cities are Belgrade (3241), Novi Sad (502), and Niš (433) in occupants per square kilometer. In Serbia, the average population density level is 91 people/km^2^. Pirot (45), Zaječar (53), Sombor (68), Kikinda (72), Kraljevo (80), and Zrenjanin (90) are the towns with low average population densities. The comparison of average population density between the towns and municipalities of Serbia reveals that the former have a twice higher density than the latter—i.e., 186 compared to 73.1 occupants per square kilometer. Officially, the lowest proportion of unemployed persons is recorded in Belgrade (6.3%), followed by Subotica (6.39%), Užice (6.43%), and Zrenjanin (6.51%) [51].

Around 5000 disasters occurred in Serbia from the 1970s to 2002 [52]. According to statistics from UNOCHA’s Reliefweb, the most common types of disasters were floods, with fifteen catastrophic floods occurring between 1988 and 2014. In Serbia from 2007 to 2016, about 20 disasters took place, killing 90 people, injuring 620 people, leaving 1470 people homeless, and causing material damage amounting to 2 million dollars [53]. Serbia is located in an area of moderate seismic activity in terms of the number, frequency, and magnitude of earthquakes. It also features an uneven distribution of epicenters, making it difficult to discern seismically active faults [54]. Stronger-intensity earthquakes (intensity of VIII–IX) were recorded at the following locations from 1900 to 1970: Rudnik, Lazarevac, Juhor, Krupanj, Jagodina, and Vitina. Only three moderate-intensity earthquakes were recorded in the following locations from 1970 onwards: Kopaonik (a mountain), Mionica, and Trstenik [55].

Between 1915 and 2013, 848 occurrences of flooding were recorded, resulting in 133 deaths [56], with the most critical event occurring in May 2014. The most significant watersheds were experienced in Kolubara (1996 and 2011); Great Morava (1999); Kolubara and Drina (2001); South Morava (2007); West Morava, Drina, and Lim (2009); Great Timok (2010); Pčinja (2010); and Drina (2010) [57,58]. Dragićević et al. [25] found the following area sizes to be vulnerable to natural hazards in Serbia: seismic hazards of VIII–IX on the Mercalli–Cancani–Sieberg scale (MCS), 16,388.59 km^2^ (18.55%); seismic hazards of IX–X on the MCS, 1109.71 km^2^ (1.26%); excessive erosion, 3320.80 km^2^ (3.76%); landslide hazards, 13,327.60 km^2^ (15.08%); vulnerable to drought, 18,306.93 km^2^ (20.72%); potential flooding, 15,198.07 km^2^ (17.20%); at highest risk of forest fires, 3154.95 km^2^ (3.57%); and total size of vulnerability in Serbia, 50,659.87 km^2^ (57.33%). Moreover, the territory of the Republic of Serbia has been affected by different epidemics: plague has occurred on several occasions (in the years of 1348, 1362, 1428, 1430, and 1438); during the First World War, the Serbian army and public were greatly affected by typhus; there was a smallpox epidemic in Yugoslavia in 1972; there were tularemia outbreaks during the 1990s; the COVID-19 pandemic struck during the years 2019–2021 [5]. Additionally, 16,357.13 ha of forests was burned (853 in total) between the years 1999 and 2008. Aleksić et al. (2009) [59] found that the total damage caused amounted to 33.56 billion dinars, the average area burned per forest fire was 19.18 ha, surface fires accounted for 85.68% of the burnt area, and crown fires accounted for 14.32 percent. The regions most at risk of these fires are east and southeast Serbia.

### 2.2. Basic Characteristics of Local Self-Governments

**Figure 3:**
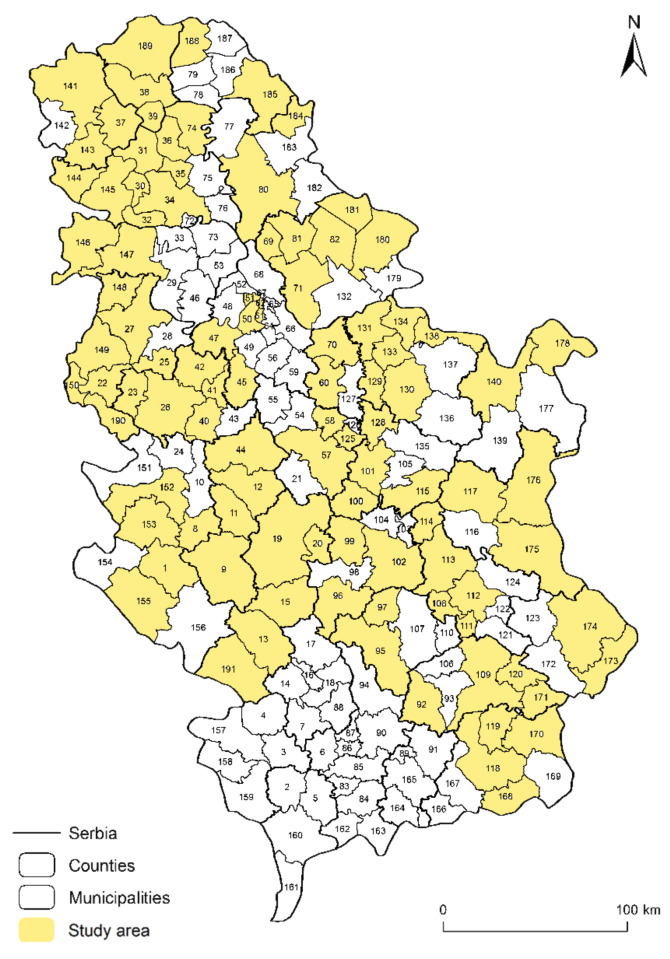
Study areas.

This survey included 105 local governments in the Republic of Serbia, of which 79.05% were for municipalities and 20.95% were for towns (Figure 3). The complete table of all Serbian municipalities (with IDs) is shown in the Supplementary Materials. Of these, 74.29% were allocated budget funds for DRM, while 25.71% were not. In terms of disaster regulations, only 29.52% have fully adopted appropriate regulations, while 35.2% have only carried out disaster risk assessments.

The majority (97.14%) of these local governments have disaster headquarters and did not complete a disaster risk assessment document (65.09%). Furthermore, 82.86% have cooperated with relevant procedures in the DRM field, while 53.33% have not taken into account the needs of vulnerable groups. The majority of local self-governments help communal companies in different phases when disasters strike (Table 1).

### 2.3. Questionnaire Design

The structured questionnaire was developed using closed-ended five-point Likert scale questions (where 1 means strongly disagree and 5 means strongly agree). The questionnaire encompassed the following aspects of disaster risk management: (1) degree of preparedness and legal framework, (2) financial framework for DRM, (3) disaster vulnerability assessment and protection and rescue plans, (4) disaster response headquarters, and (5) cooperation and communication in the context of DRM. The questionnaire was answered by 17 out of 23 mayors from July to September 2018 (Appendix A). As for heads of the local disasters sector (first target group), a unique questionnaire was compiled based on the competencies of local self-governments in the field of disasters as regulated by applicable laws, as well as on the means of financing and coordinating DRM entities (Supplementary Materials). Several published survey approaches were consulted [60,61]. Our quantitative analysis was compatible with the Helsinki Declaration [62], which defines the standards for sociomedical research concerning human subjects. Participants provided their informed consent to participate in the study. The research protocol was approved by the committees of the Institutional Review Board, University of Belgrade and the Institutional Review Board of Scientific-Professional Society for Disaster Risk Management and International Institute for Disaster Research (protocol code 001/2021, 15 June 2021).

### 2.4. Analyses

Descriptive statistics were calculated for all research questions (Appendix A and Supplementary Materials). Chi-square tests [63] were used to examine the relationship between the disaster management units’ knowledge of regulations and the observed variables. In addition, *t*-tests [64] were used to examine the relationship between the towns and municipalities of Serbia with regard to the following variables: general preparedness, awareness of the law on disaster risk reduction, funds allocated from the local budget, and communication assessment results.

A multivariate regression analysis was used to identify the extent to which total scores for the main dependent variables (preparedness of the local self-government; obligations of the local self-government; support scores received) were associated with the independent variables (budget funds; cooperation; disaster risk assessment; protection and rescue; the assessment of legislation; headquarter preparedness) (Table 2). We tested our central hypothesis, which related to the extent to which budget funds for DRM could predict the capacity development of local self-governments. Previous investigations on the residual scattering diagram [65] revealed that the assumptions of normality (normal probability plot P-P and scatterplot), linearity, multicollinearity (*r* = 0.79), and variance homogeneity were not violated. The internal consistency of Likert scales for the attitudes of the heads (mayor) of disaster sectors in local self-government units (eight items) was good, with a Cronbach’s alpha of 0.84. The mentioned statistical analysis was performed using IBM SPSS Statistics, Version 26.

## 3. Results

The study’s findings are presented in three dimensions:

- Predictors of the local self-government for the capacity development of local self-governments for DRM;
- Attitudes of the employees of the DRM sector in local self-government units;
- Attitudes of the heads (mayor) of disaster sectors in local self-government units.

### 3.1. The Predictors of the Capacity Development of Local Self-Governments for Disaster Risk Management

The multivariate regression analyses showed that the most important predictor of local self-government preparedness for a disaster is the assessment of the legislation (*β* = 0.150), which explains 2.13% of the variance in the score. All the other variables did not have significant effects on the score. This model (R^2^ = 0.59, Adj. R^2^ = 0.56, *F* = 20.11, *t* = 34.19, *p* = 0.000) with all the mentioned independent variables explains 56% of the variance of local self-government preparedness for a disaster (Table 2).

**Figure 4:**
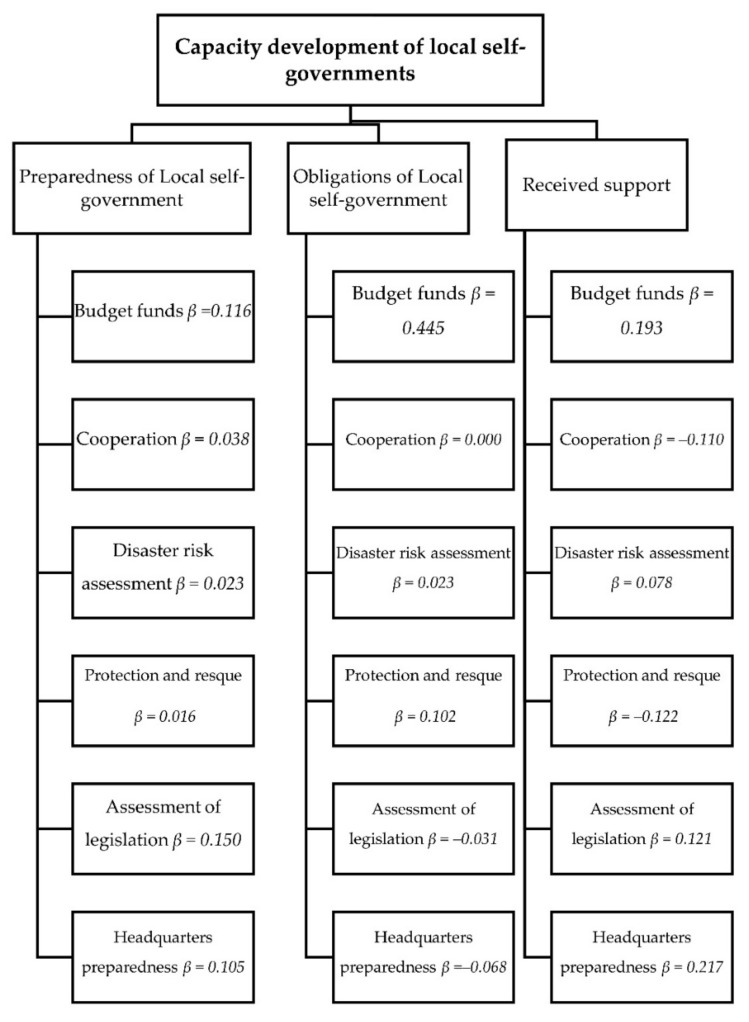
The predictors of the capacity development of local self-governments for DRM.

Further analysis showed that the most important predictor of the level of information regarding the obligations of the local self-government that arose from the current disaster law is having funds for disaster risk management in their budget (*β* = 0.445), which explains 18.92% variance in the score. Other variables did not have significant effects on the score. This model (R^2^ = 0.23, Adj. R^2^ = 0.17, *F* = 20.11, *t* = 4.15, *p* = 0.000) with all the mentioned independent variables explains 17% of the variance of the information level of local self-government obligations that arose from the current disaster law. The multivariate regression analysis showed that not all the variables had significant effects on receiving support for improving DRM (Table 2 and Figure 4).

More analyses found that there was a slight relation between local self-governments’ budget funds for DRM and the preparedness of the local self-government (*r* = 0.232, *p* = 0.019) and the information level of local self-government obligations that arose from the current disaster law (*r* = 0.444, *p* = 0.000). It can be said that with the growth in the budget of local self-government, the readiness and information level of local self-government obligations increases. There was no statistically significant correlation between the budget of local self-governments and receiving support and headquarters preparedness (Table 3). Additionally, we could not find a statistically significant correlation between disaster risk assessment, protection and rescue, rules of procedures, and the dependent variables. Further analysis showed a statistically significant correlation between the annual work plan and preparedness (*r* = −0.205, *p* = 0.041) (Table 3). A local self-government that does not have an annual work plan has a lower level of disaster preparedness. Additionally, we found a statistically significant correlation between headquarters’ preparedness and the assignment of duties to members of the headquarters (*r* = 0.222 *p* = 0.026). It was determined that with the increase in the level of assignment of duties to the members of headquarters, the level of readiness of the headquarters increases.

### 3.2. Attitudes of the Employees of the Disaster Risk Management Sector in Local Self-Government Units

#### 3.2.1. Degree of Preparedness and Legal Framework

**Figure 5:**
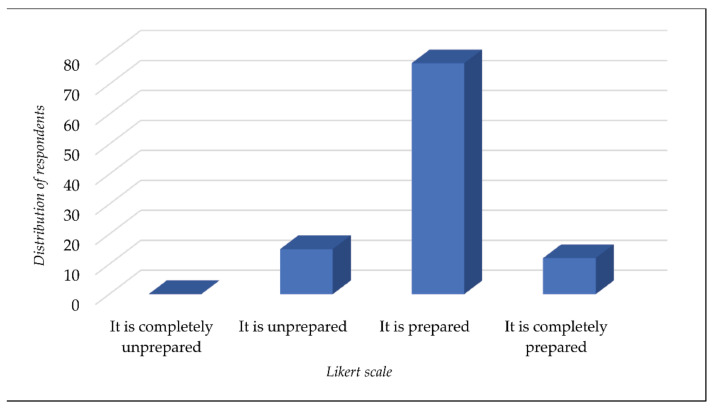
Preparedness (Likert scale) of the local self-government in Serbia for disaster events.

In total, 5% of employees of local DRM units assessed their local self-government as insufficiently prepared to deal with a disaster event, 80% deemed them to be averagely prepared, and 15% deemed them to be fully prepared (*x* = 2.97; sd = 0.51) (Figure 4). None of the respondents assessed local self-governments with different socioeconomic characteristics (Appendix B) to be completely unprepared for disasters. The application of a *t*-test for independent samples showed that, although the readiness rates of town communities to respond to disasters were generally assessed to be more favorable than those of municipalities, there was no statistically significant difference between the two (Table 4 and Figure 5).

In the survey, the respondents were asked to indicate, in their personal opinions, the major problems with and obstacles to increasing the capacity of their local self-government units in terms of DRM. The majority (over 70%) specified the lack of finances as one of the most serious problems faced by risk management. More than 40% of the respondents highlighted problems with human resources—i.e., the lack of fully qualified and suitably experienced staff to complete the necessary tasks—while more than 10% of the respondents additionally singled out the lack of equipment and legislation issues (imprecise regulations, data inconsistency, devaluation of existing regulations by announcing new ones, etc.). In addition, the respondents in the survey also stated that there was poor coordination between the system entities and citizens, resulting in citizens lacking education on the matter of DRM. The capacity analysis suggested that there were no differences in level across Serbia—i.e., these problems were universal across towns and municipalities, with the size of settlements being an irrelevant factor.

Regarding the interviewees’ awareness of the obligations of local self-governments based on the law on disaster risk reduction, 10% of respondents stated that they were keenly aware of it, while the rest (90%) stated that they were fully aware of the obligations of the local authorities. No respondents stated that they were either insufficiently acquainted with or unacquainted with this issue. The results obtained suggest that there were statistically significant differences between towns and municipalities, whereby the urban population (town residents) was significantly more acquainted with the legal framework than those at the municipality level (Table 4).

Further analyses show the following activities carried out by local self-governments in the territory of Serbia regarding DRM (% of local self-governments) (Table 5).

Regarding the documents above, it was only in the annual work report that the chi-square test implied there was a statistically significant difference between towns and municipalities, which was in favor of the latter (*X*^2^ = 6.19, *p* < 0.05). Except for the mentioned document, which was predominantly adopted by municipalities, the difference in the proportion of completion of the regulatory documents (decisions, reports, plans, schemes, etc.) was not significant. The share of towns in which a threat assessment was conducted and protection and rescue schemes were created was alarmingly low. Additionally, the interior ministry issued permits for disaster risk assessment and rescue schemes only at rates of 23.5% and 34.8%, respectively.

The respondents in the survey also assessed the law in power. Some 30% of the respondents assessed it as fully regulating disaster management, while 70% of the participants in the survey found it to be inadequate. Within the frame of the topic, the chi-square test showed no significant differences between respondents from towns and those from municipalities (*X*^2^ = 0.08, *p* > 0.05). The respondents who assessed the law to be fully appropriate described it as precisely defining the competences and assignments, with some of them observing that the law had not been fully implemented, thus preventing them from perceiving it accurately. On the other hand, the respondents who assessed the law as inadequate emphasized its inconsistencies with other laws, pointing to the large number of competencies transferred to local self-government units as well as the rather unspecified role of all-purpose civil protection units, the unclearly defined rights and obligations of members of all-purpose civil protection members, the lack of instructions for the application of laws and bylaws, their inapplicability in the field of civil protection, etc.

**Figure 6:**
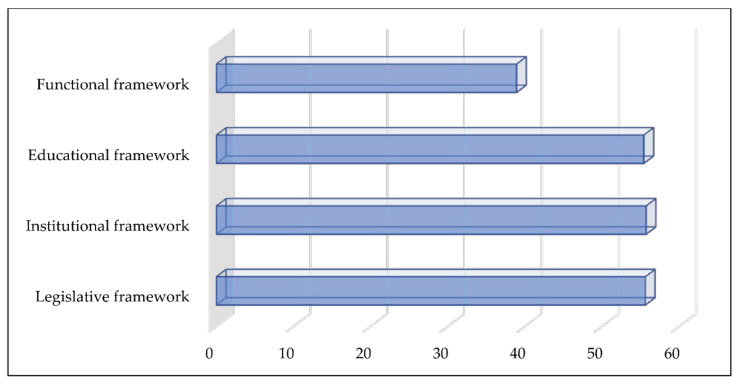
Forms of assistance needed to improve local regulatory acts or compose them appropriately.

In the survey, the respondents listed their needs for assistance as follows: the legislative framework (decisions, rule of procedure, instructions, etc.), 55.6%; institutional framework (setting up services, departments, or special organizations; the formation of civil protection units, etc.), 55.7%; educational framework (appointing qualified staff, providing courses, issuing licenses, education, workshops, round tables, public debates, etc.), 55.4%; functional framework (the lack of understanding on the part of the authorities, the inability to put acts into force, unqualified staff, the lack of equipment, etc.), 38% (Figure 6).

As additional forms of assistance, the respondents specified the type of support required from the sector in the area of prevention—i.e., drafting acts and more effective communication. The results of the chi-square test suggest that there were significant differences in the assessment of the need for legislative and educational assistance (*X*^2^ = 3.78, *p* = 0.05; *X*^2^ = 4.56, *p* < 0.05, respectively).

#### 3.2.2. Financial Framework for Disaster Risk Management

Concerning allocating funds from local self-governments for risk management and risk reduction, all the respondents from local self-government units in areas with town status answered in the affirmative. The chi-square test was used to examine the significance of the differences between municipalities and towns, with the resulting differences being significant (*X*^2^ = 6.90, *p* < 0.05). The respondents were also asked to specify the nominal amount and funds (%) allocated from the local budget in the previous fiscal year. It was found that about 70% of the respondents from towns were acquainted with the amount allocated, though only half of them could present the amount in a percentage. The allocated funds in the towns ranged from 500,000 RSD to 168,920,940 RSD (1 USD = 99.5 RSD) (M = 22,517,246; SD = 40,959,620), with the percentage ranging from 0.1 (Sremska Mitrovica, Zrenjanin, Užice) to 1 (Šabac). Statistically, the percentage of funds allocated to towns and municipalities was not significantly different, although the average values point to greater amounts being allocated to municipalities. However, when interpreting these results it is important to note that a large number of respondents (half of the representatives of local self-government units and more than half of the representatives of municipalities) gave no response to the questions, which implies that these figures do not reflect the actual situation. Additionally, the statistical significance of this test is close to the borderline, with these differences being referred to as potentially significant (Table 4).

**Figure 7:**
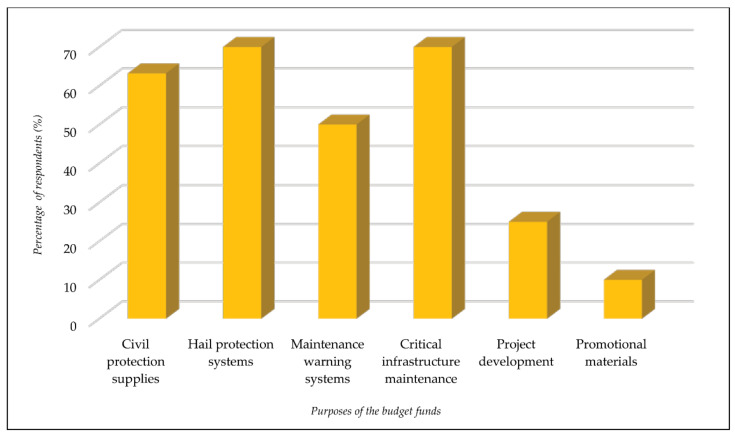
Allocating funds from local self-governments for DRM and risk reduction.

Regarding the purposes of the budget funds above, the responses were as follows: civil protection supplies (63.2%); hail protection systems (70%); maintenance of emergency population warning systems (50%); water streams and critical infrastructure maintenance (70%); project development (25%); promotional materials (10%) (Figure 7).

In addition to the responses offered by the survey, the respondents additionally stated that the funds were used for the procurement of equipment; fire truck fuel; and miscellaneous purposes—e.g., maintaining open canals. In this respect, the comparison between towns and municipalities reveals significant differences in the financing of civil protection (*X*^2^ = 6.14, *p* < 0.05), hail protection systems (*X*^2^ = 5.94, *p* < 0.05), and urban protection services (*X*^2^ = 3.78, *p* = 0.05), whereby the amounts allocated were higher in towns, as reported by town representatives from all the areas surveyed.

Compared to municipalities, a significantly greater number of representatives of local self-government units in towns were informed about the available funds/international funds (*X*^2^ = 4.604, *p* < 0.05), which still does not imply that they possessed deep knowledge about the funding, given that only half of the respondents from towns were considered to be informed. Only eight out of the total 23 respondents said that they applied for some of the funds from organizations such as the EU IPA Cross-Border Cooperation funds, the UNDP office, the Regional Environmental Center for Central and Eastern Europe (REC), the Republic Construction Commission, the Embassy of Japan, and the World Bank.

#### 3.2.3. Disaster Vulnerability Assessment, Protection and Rescue Plans

Respondents employed in local self-governments that had undertaken vulnerability assessments and developed protection and rescue schemes were asked whether or not their local self-governments had undertaken any actions to improve the quality of town planning schemes—e.g., disaster response schemes or communal company action plans. Some 43.8% of respondents answered in the affirmative, which was not significantly different from the percentage obtained from municipalities (*X*^2^ = 2.19, *p* > 0.05). The question of “Have the communal companies developed vulnerability assessment and protection and rescue schemes?” was answered by only one third of the respondents. Only in two towns (Pančevo and Užice) did the respondents answer that all the communal companies had developed the schemes mentioned above, while the others specified that companies either had or were in the process of developing them, or that they had not been designed at all.

In addition to the communal companies, hospitals and related health institutions—e.g., veterinary hospitals or Red Cross organization units; the Institute of Transportation CIP; the Welfare Department; TV stations; fire brigades; citizens’ associations; qualified legal entities in the realm of protection; public and private companies; academic institutions, etc., were identified in the survey as crucial to disaster management. Only 8.7% of respondents said that vulnerability assessment and protection and rescue schemes had been developed by some of the institutions and organizations above; some 26% stated that they had not been developed, while other respondents gave no response.

When asked if they had received any support in the process of drafting protection and rescue schemes and vulnerability assessment plans, only 21.7% of respondents answered in the affirmative, 30.4% said no, and others gave no answer. In this assessment, the difference between towns and municipalities was not statistically significant (*X*^2^ = 0.121, *p* > 0.05)—i.e., a small number of representatives from local self-government units from both towns and municipalities reported to have received support in designing these schemes. When asked to specify the type of support received, respondents reported that they had received it from the institutional domain (two respondents)—i.e., the disaster management sector; the educational sector of the activities of the National Training Center and Safe Serbia association (three respondents); and the functional domain (two respondents).

Assessing the support received, 26.1% of the respondents rated it as insufficient, 17.4% rated it as relatively sufficient, and only one respondent (4.3%) assessed it as sufficient. The majority of the respondents (more than 50%) gave no answer to this question; however, the comparison between the responses obtained from towns and municipalities does not identify any statistically significant differences (Table 4).

#### 3.2.4. Disaster Response Headquarters

The respondents were queried as to whether or not a disaster response headquarters had been established in their local self-governments, and, if so, whether or not they were familiar with its competencies. Statistically, there was no significant difference between the former and the latter—i.e., *X*^2^ = 0.49, *p* > 0.05, and *X*^2^ = 1.81, *p* > 0.05, respectively. Respondents were then asked to answer the question “Are disaster response headquarters prepared to respond to an emergency?”. In total, 55% of respondents from towns cited that the disaster response headquarters were fully prepared to adequately respond to disasters, while 45% reported that it was partially prepared. In this respect, the *t*-test for independent samples showed no statistically significant difference between the respondents from the two groups (*t* = 0.50, *p* > 0.05).

The question “Did the disaster response headquarters or administration unit establish communication with relevant disaster response-related institutions in your town?” was answered in the affirmative by 90% of respondents, which did not differ significantly from the responses given by the local self-government representatives from municipalities (*X*^2^ = 0.29, *p* > 0.05). Establishing communication primarily involves the following aspects: the election of staff members, the adoption and harmonization of acts, decision making, coordination aimed at implementing staff activities, the implementation of conclusions, making orders and recommendations, taking prevention measures during disasters, communicating information to the population, providing support and training, organizing meetings and workshops, providing material resources. The results reveal that there was relatively good (70%) or very good (30%) communication, as estimated by the respondents in the survey. Statistically, the estimates of representatives from the first group (towns) were not significantly different from those obtained from the second group (municipalities).

#### 3.2.5. Cooperation and Communication in the Context of Disaster Risk Management

The following structured enquiry asked the respondents to assess the extent to which they established cooperation with neighboring municipalities over the past few years. Some 42.1% of respondents from towns assessed it as considerable, 36.8% to a lesser extent, while 21.1% opted for the absence of cooperation. No statistically significant difference was obtained between the two groups of local self-governments representatives (*t* = 0.39, *p* > 0.05). Based on the respondents’ answers, the cooperation included exchange of experiences, relief supplies, providing volunteers and logistical support, communication on legal obligations issues, hiring experts for a damage assessment team, etc.

When asked to assess the cooperation with neighboring municipalities in disaster prevention and management, only 10% of respondents from towns cited that the cooperation was considerable, 45% described it as poor, while 45% of the respondents reported on the absence of any cooperation. The application of the *t*-test for independent samples, no statistically significant differences were found between the two groups queried (*t* = −0.994, *p* > 0.05) (comparative results are presented).

When asked to identify the form of cooperation in each of the cooperation fields, 16.7% of the respondents specified the planning and development of joint projects aimed at financing disaster prevention and management systems, 6.6% cited the planning and development of joint projects intended for financing mitigating aftermath consequences, and 27.8% stated joint training. Besides the domains offered by the questionnaire, the respondents also reported the exchange of experiences as a form of cooperation, as well as some situation-specific cooperation.

The percentage of affirmative answers in the areas above was not significantly different between the respondents from local self-governments units and those from municipalities. Within this survey item, 40% of respondents implied the existence of cooperation with cross-border municipalities, while 60% reported that there was no cooperation at all. The chi-square test suggested no significant differences between municipalities and towns (*X*^2^ = 3.11, *p* > 0.05).

**Figure 8:**
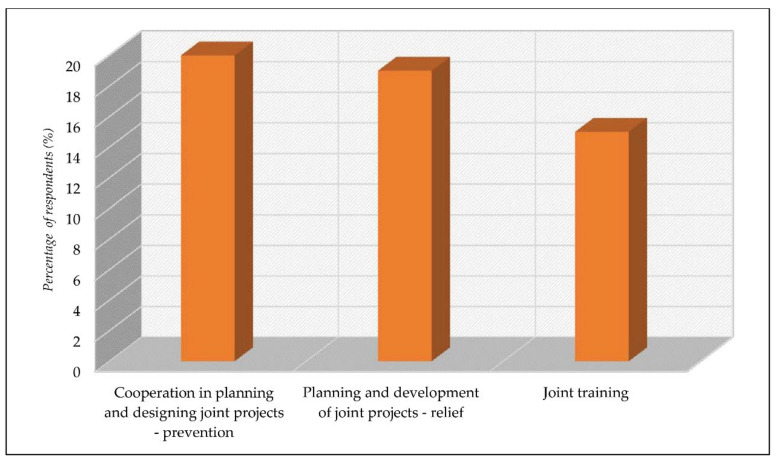
Overview of cooperation with cross-border municipalities regarding DRM.

Regarding cooperation with cross-border municipalities, the respondents described it as (a) cooperation in planning and designing joint projects aimed at financing prevention and emergency management systems (20%), (b) the planning and development of joint projects intended to finance relief in the aftermath (15%), and (c) joint training (15%) (Figure 8).

Within the assortment of questions regarding cooperation, the respondents were asked if they had cooperated with governmental institutions in charge of disaster risk prevention. Some 95% of the representatives of local self-government units answered in the affirmative. No statistically significant differences were obtained between towns and municipalities either in the existence of cooperation with government institutions (*X*^2^ = 0.86, *p* > 0.05) or in forms of cooperation with the institutions above. Some 90% of respondents from towns had experienced the cooperation with the local district disaster response headquarters. The respondents in the survey reported on cooperation within legislative (35%), institutional (25%), educational (35%) and functional fields (65%). Additionally, the collaboration included data and information exchange, joint meetings with local self-government headquarters, and coordination during disasters.

Some 85% of respondents from towns cited that citizens and the wider community were involved in prevention activities, mostly during disaster events and in the aftermath period, in meetings in local community centers (73.7%). The citizens also participated in educational workshops (21.1%), activities related to vulnerability assessment and protection and rescue schemes (15.8%), and civil protection training and disaster drills (10.5%). The participation of citizens was the lowest in forums, which were organized in one town only. There was no significant difference between towns and municipalities with respect to the participation of citizens in the activities above (*X*^2^ = 0.89, *p* > 0.05).

The question “Do you consider local administration employees and officials well informed about disaster prevention and management?” was answered in the negative by 57.9%, and by 42.1% in the affirmative. The greatest proportion of the latter believed that improvements could be made through education, which unfortunately had been systematically and unjustifiably neglected. It is for this reason that more than 50% of representatives proposed education in the respective field as the most viable proposal for raising public awareness of disasters. On the other hand, when the knowledge of the citizens was in the focus of the query, as much as 85% of respondents answered in the negative. Statistically, the obtained results were not significantly different compared to municipalities (*X*^2^ = 1.44, *p* > 0.05). Some 80% of respondents from towns believed that the knowledge could be improved through the educational framework (workshops, forums, public debates), while 45% of respondents considered the institutional framework the most useful to that end. Some of the offered answers were the introduction of subjects in primary schools and raising the awareness of citizens regarding civil protection by governmental bodies.

Within the assortment of questions relative to priorities in raising awareness on disaster protection, and the prevention, as many as 47.4% of the respondents highlighted the essential role of political authorities in local self-governments, representatives of local media followed, as well as local self-government employees, managing boards of public companies and institutions, and finally activities of school children, the youth, and citizens. Based on the responses in the survey, the civil society organizations were most involved in the preparation of risk assessment and protection and rescue schemes, as well as in long-term improvement and development plans (27.8%), while only two respondents reported the involvement of the Commission for Gender Equality and the related subjects, and one person from the Women’s Association. A statistically significant difference was obtained only regarding the involvement of civil society organizations (*X*^2^ = 5.03, *p* < 0.05), in favor of towns. As for the local self-government units which failed to complete the related documents, the greatest proportion of respondents believed that civil society organizations have the potential to contribute in the field, along with the Commission for Gender Equality/officials within that domain. Only four respondents believed that Women’s Associations could contribute to the domain. No significant differences between towns and municipalities were observed in any of the options above. The respondents were also queried about the extent to which the needs of vulnerable groups, such as the Roma, people with special needs and those with disabilities, etc. were observed. Slightly more than half of the respondents answered the question (56.5%), whereby 58.8% considered that the needs of the population was fully observed, while 23.1% respondents stated that they were only partially taken into account or not considered at all.

Concerning meetings, regular meetings were mostly held quarterly (in about 70% of towns), and emergency ones were held when needed. In Leskovac, headquarters members meet only once or twice a year; in Pancevo, these were run six to eight times a year; and in Nis, in 2016 as many as 14 sessions were held. It was only in Kragujevac that professional and operational teams were not formed, while the number of teams set up in other towns ranges between three (Loznica, Novi Sad, Uzice, and Sabac) and 23 in Sombor. The analysis conducted in 2014 showed that the average number of professional and operational teams was five in towns (M = 4.86), even amounting to seven in 2017 (M = 6.86); the *t*-test for the dependent samples revealed no significant difference.

The results suggest a change insofar as legal subjects have been trained in all the towns—i.e., 11 in Jagodina, 73 in Sombor, and as many as 66 in Belgrade. The analysis also revealed that by 2014 only in 12 towns Civil Protection Commissioners and Deputy Commissioners were appointed. In Belgrade, the legal subjects were appointed only in suburban municipalities. In this respect, in 2017, it was only in Novi Sad that Civil Protection Commissioners and Deputy Commissioners were not appointed, other towns having met the regulation. In addition, the data from Smederevo indicate that the commissioners were appointed, however, their replacement was recommended. According to the analysis conducted in 2014, only seven towns (Vranje, Jagodina, Kruševac, Niš, Novi Sad, Požarevac, and Sombor) had civil protection units set up; however, the number amounted to 11 in 2017, with the newly included towns being Čačak, Sremska Mitrovica, Kragujevac, and Kraljevo. In Uzice, civil protection units were formed only temporarily. The very existence of civil protection units does not imply that they are equipped or trained. The exceptions are in Kraljevo, Sombor, and Sremska Mitrovica, while in Uzice and Cacak the civil protection units are only partially trained. The procurement of equipment is underway in Kragujevac, and the training of civil protection units is planned for the fall of 2017. We have not received the information on the exact capacity of the facilities; however, the available data imply that these range from 40 m^2^ in Uzice (which is less than in 2014 (100 m^2^) to 2,300,000 in Belgrade, which correlates with the size of the town and the population.

The testing of the disaster management system through disaster drills is only occasionally and unsystematically organized in fewer than half of the towns (45.5%). The disaster drills are most commonly performed in Leskovac (twice or thrice a year), Kragujevac (twice a year), and Pancevo (once or twice a year).

Regarding emergency population warning systems, the analysis showed that the system is being modernized in Belgrade and Valjevo, while it was reported that in Zrenjanin, Pancevo, Loznica, Sombor, and Uzice the system is in satisfactory condition. In Jagodina, Kragujevac, and Krusevac, the system is operational; in Novi Pazar, the maintenance standards are not met. In all other towns, the sirens are poorly maintained and faulty. Sirens were installed in Serbia almost 50 years ago, and it can be said that their functionality is largely questionable and local governments have a legal obligation to complete acoustic studies within 3 years. The results of the assessment also report on insufficient acoustic coverage—e.g., in Nis and Leskovac. Additionally, the acoustic studies in local self-governments are outdated and do not meet the requirements of settlements whose area increased over time. The analysis carried out in 2014 reported on the plans for developing new acoustic studies in Sremska Mitrovica and Čačak. In 2017, these were underway in Sremska Mitrovica and they were fully realized in Čačak, with the modernization of the equipment being in progress. Regardless of the explicit need for modernized acoustic studies, these have not been developed so far.

In contrast to the year 2018, when the telecommunications, information, and communication technology systems that enable and support the operation of disaster management systems were assessed as rather outdated, some positive movements have been observed lately. In this respect, the majority of towns have reported favorable or satisfactory changes. In Novi Pazar, the equipment is in solid condition, whereas the reports from Novi Sad, Smederevo, Valjevo, and Nis indicate unfavorable conditions relative to the equipment, suggesting the need for modernization.

Regarding individual and collective protection, three years after the initial analysis, in Belgrade, the conditions changed from ‘alarming’ to ‘satisfactory’. The results suggest incomplete and outdated means of individual and collective protection, and poor general condition, the only exception being the town of Uzice, which was described as “partially equipped” but still in an unsatisfactory condition. This year’s analysis infers somewhat more favorable circumstances, with slightly more than half of the reports assessing the situation to be good or satisfactory. Inspections are being conducted in Smederevo, Pančevo, Novi Pazar, and Niš; the report from Valjevo described the equipment as ‘satisfactory’ but assessed the citizens as untrained for individual and collective protection. The report from Jagodina is similar, implying that the individual and collective training schemes have been designed and developed but not realized in practice.

The assortment of questions that follows concerns disaster prevention and the operation of local self-government sectors under normal circumstances. Respondents from local self-governments in towns who participated in the development of vulnerability assessment and protection and rescue schemes were asked if their local self-government had taken any steps to enhance quality of town planning schemes or to improve the disaster response and communal companies action schemes during disasters. A total of 43.8% of respondents answered in the affirmative, which was not significantly different from the responses obtained from local self-government units in areas with the status of municipalities (*X*^2^ = 2.19, *p* > 0.05).

Only one third of the respondents in the query answered in the affirmative to the question related to the development of vulnerability assessments and protection and rescue schemes in the local community. The respondents from Pančevo and Užice cited that all the communal companies had developed schemes, while the remaining proportion of respondents either listed the companies who had developed schemes, denied the existence of the schemes, or stated that they were in the drafting stage. Besides communal companies, institutions perceived by the respondents in the survey as crucial to disaster prevention and management were as follows: hospitals and related health care institutions, veterinary hospitals, the Red Cross Organization, the Institute of Transportation CIP, protective services, TV stations, fire brigades, citizens’ associations, protection-qualified legal entities, public and private companies, academic institutions, etc. The question “Do these institutions have vulnerability assessment and protection and rescue schemes developed?” gave the following results: Only 8.7% of respondents claimed that some of the institutions had the schemes developed, 26% stated the schemes did not exist, and the remaining proportion of respondents in the survey did not answer. The query reported that threat assessment has been undertaken in about 36% of towns, whereas protection and rescue schemes were developed in 16.7%, which is alarming, as these documents are the building blocks for the prevention activities that include measures and activities to be taken during disaster events and resource allocation, organization. and coordination at the local level, which are all essential for the implementation of competencies prescribed by law financially, operationally, and institutionally.

The results of the chi-square test revealed a correlation between the information gathered from local self-government representatives and the following variables examined: the decision passed on the formation of the headquarters (*p* = 0.050); duties assigned to headquarter members (*p* = 0.028); the assessment of legislation in power in the domain of disaster management (*p* = 0.022); budget financing (*p* = 0.05); the steps taken to enhance the quality of town-planning schemes (*p* = 0.050); establishing disaster management headquarters (*p* = 0.050); the assessment of the preparedness of disaster management headquarters (*p* = 0.050); the assessment of the communication established between the disaster management headquarters and relevant subjects (*p* = 0.01); cooperation with governmental institutions in the domain of disaster prevention (*p* = 0.43) (Table 6).

On the other hand, the results of the chi-square test showed that there was no correlation between how informed local self-government representatives were and the following variables encompassed by survey: the rules of procedure were adopted; an annual work report was adopted; an annual work plan was adopted; a decision was made on the formation and operation of civil protection; a decision was passed on the establishment of a civil protection unit; a conclusion was made regarding the appointment of the civil protection commissioner; a risk assessment team was set up; risk assessment and protection and rescue schemes and flood defense schemes were adopted; insight was gained into international funds intended for improving readiness to respond to disasters; support was given in developing schemes; insight was gained into the competencies of disaster headquarters; an assessment of cooperation with other municipalities during disasters was undertaken; an assessment of cooperation with other municipalities in the disaster prevention domain was undertaken; an assessment of cooperation with cross-border municipalities in the disaster prevention domain was undertaken; an assessment of the needs for cross-border cooperation in the domain of disaster management was undertaken; involving citizens in disaster prevention framework (Table 6).

### 3.3. Attitudes of Heads (Mayor) of Disaster Sectors in Local Self-Government Unit

The obtained results show the following mean values of the mayor’s agreement with the views:

(a) The legal solution according to which the mayor is the commander of the disaster headquarters is good and should not be changed (*X* = 4.8);
(b) The city administration with all sectors is fully prepared to respond to disasters (*X* = 4.2);
(c) The competencies of local self-governments in disaster management in Serbia are fully and sufficiently precisely regulated by laws and bylaws (*X* = 3.8);
(d) The competences that the city has in disaster management are sufficiently implemented (formed operational expert teams, appointed commissioners of civil protection and their deputies, formed and equipped and trained civil protection units, situation center, means of alert, etc.) (*X* = 4.1);
(e) Strategic risk assessment plans provided by law are adopted and implemented in cities sufficiently and in a timely manner and the style of disaster management is mostly proactive (*X* = 3.8);
(f) Actors in local self-government (public administration, public services and policy makers, citizens) are sufficiently trained and educated on disaster management (*X* = 3.6);
(g) Activities and voluntary engagement of citizens in emergency situations are appropriately regulated by laws and bylaws and appropriate standard procedures (*X* = 3.7);
(h) Civil society organizations, citizens’ associations, and volunteers are very important in disasters (*X* = 4.8) (Table 7). Thus, the highest values were recorded in the attitude related to the legal solution, while the lowest values were recorded in the attitude related to training and education. On the other hand, observed in relation to the city, the lowest total mean values of attitudes were recorded in the mayor from Belgrade (*X* = 3.3) and the highest in the mayor from Sremska Mitrovica (*X* = 4.5).

About 47% of mayors agreed that the legal solution according to which the mayor is the chief of staff for disasters is good and should not be changed. When assessing the accuracy of regulating the competencies of local self-government by laws and bylaws, mayors gave more diverse answers, and 82.4% agreed with the above statement. When asked about the extent to which the competencies of the city have been implemented in disasters, 35.3% of them answered that they completely agreed, 58.8% agreed, and 5.9% declared themselves undecided. To the statement “Strategic risk assessment plans provided by law are sufficiently and timely adopted and implemented in cities, and the style of disasters management is mostly proactive”, one gave the answer “I completely agree”, while 64.7% gave the answer “I agree” and three were undecided. No mayors disagreed with this statement. Diverse answers were also received to the question on educating actors in local self-government about disasters, with only three mayors fully agreeing that actors are sufficiently educated. It is interesting that more than half of the respondents from local self-government units (57.9%) pointed out that employees and officials in the city administration are not sufficiently informed about prevention and emergency management, and that the greatest improvement could be achieved through the educational framework.

**Figure 9:**
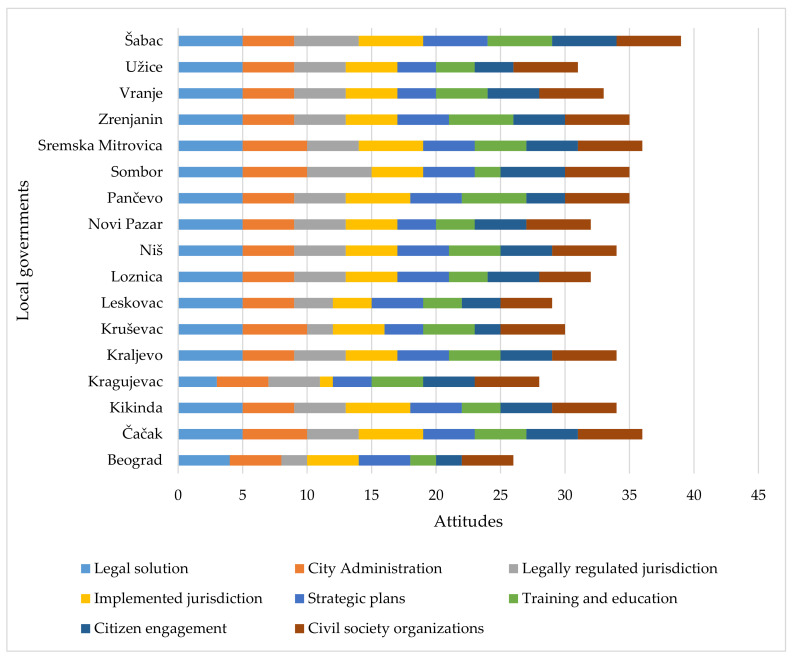
Attitudes of heads (mayor) of disaster sectors in local self-government units.

Only 11.8% of respondents completely agreed, 58.8% agreed, 17.6% were undecided, and 11.8% disagreed with the statement “Activities and voluntary engagement of citizens in disasters are appropriately regulated by laws and bylaws and appropriate standard procedures”. Although they do not fully agree when it comes to the legal framework and procedures, 82.4% of mayors fully agreed with the importance of civil society organizations, associations of citizens, and volunteers in disasters, while the rest agreed. However, no significant correlation between the mayor’s assessment of the importance of citizen involvement and the assessment of local self-government units on citizens’ information was obtained (*r* = −0.310; *p* > 0.05); although all the mayors fully agreed or disagreed with the importance of citizen involvement, as many as 85% of the respondents from local self-government units answered that their information is not sufficient (Figure 9).

## 4. Discussion and Recommendations

In this study capacity development of local self-governments of Serbia was investigated for DRM. At the end of the study, it was found that the most important predictor of local self-government preparedness for a disaster is the assessment of the legislation. Also, it was seen the budget funds that arise from the current disaster law are the most important predictor of the level of information regarding obligations of local self-government. More analyses found that there was a slight relation between local self-governments budget funds for DRM and preparedness of local self-government and information level of local self-government obligations that arise from the current disaster law. At the degree of preparedness and legal framework, although the lower readiness rates of town communities to respond to disaster strikes are generally assessed as more favorable than that of municipalities, there is no statistically significant difference between these two, besides the towns have a higher point at the awareness on the law on disaster risk reduction dimension, too.

The local self-governments allocate funds for DRM and risk reduction are hail protection systems, water streams and critical infrastructure maintenance, and civil protection supplies. Of the respondents from local self-government units and those from municipalities, 40% of respondents implied the existence of cooperation with cross-border municipalities, while 60% reported on no cooperation at all in cooperation and communication in the context of disaster risk management. We also searched the dimensions of cooperation and communication in the context of disaster risk management, and the attitudes of heads (mayor) of disaster sectors in the local self-government unit in this study.

The comparison of opinions of the two groups of respondents in the survey—i.e., political decisions makers/mayors and the employees of local administrations of the disasters sector—suggests an attitude that only vaguely relates to the actual social needs in emergencies. In such an ambiguous environment, local disaster risk management can be portrayed as *bureau-politically incompetent* [28]. Local self-governments are often used for ‘lightning rod effects’ in efforts to escape the blame and shift it to lower levels of the government and prominent representatives, which can account for the dramatic differences in attitudes among mayors and respondents from local self-governments and the interior ministry in the survey. Giving socially and politically desirable answers instead of ones reflecting reality is a typical feature of uninformed politicians, but can also imply a maneuver that provides a space for retreat in the event of a possible future disaster [28]. The results obtained can be explained by the fact that many organizations around the world are faced with challenges in translating capacity development guidance into practice [9], and it is necessary to understand the local context [66] as well as the demographic and socioeconomic context [67,68]. Thus, the perceptions of mayors and local self-government employees of the issue of the capacity of disaster management systems can primarily be categorized as an impression management process in which people are unlikely to provide detailed insights into the current efforts to maximize the effectiveness of DRM [69]. It goes without saying that the voluntary engagement of citizens in disasters is neither adequately regulated by law nor operationally standardized [58]. Finally, the analysis shows that members of disaster management teams within local self-government units have neither the necessary qualifications nor proper education and training for DRM, and this could make it difficult to further improve the system [70]. Large local governments have had problems dealing with the demands of different stages of disaster management [13]. The laws that regulate the area of disaster risk management that are important for the functioning of public administration are numerous and located in different areas: emergencies, defense, state administration, local self-government, health, agriculture, water and natural resource management, etc. In order for laws to be applied, a complete and precise bylaw framework is needed at all levels of competence. It is undoubtedly the case that the competencies of different administrative levels regarding policies for DRM in Serbia are not fully or sufficiently regulated by laws and bylaws.

Our research findings are in agreement with the key shortcomings identified in the national strategy for disaster protection and rescue [32]; they relate to institutional, organizational, material, and technical deficiencies; cooperation shortcomings; lack of coordination and information availability; and a lack of human resources and education. Due to the factors mentioned, it is very important to work continuously on the improvement and development of better DRM mechanisms in order to provide the highest possible effectiveness and efficiency [71]. In addition, it is important to consider the fact that the highest effectiveness is at the community level, where specific needs are met [72]. The query points to a group of problems that exist at the level of local self-governments, which is also present in other states [13,50,71,73,74,75]. As of 2000, public policies in Serbia have to a great extent operated according to the principles and rules of the new public management, which is a limiting factor in implementing the principles of DRM in certain segments (the displacement of institutions from sparsely populated border areas to regional centers—i.e., institutions such as schools, health care institutions, and courts (which is known as ‘the optimization of institutions’)). Under such circumstances, the lowest price in public procurements is a decisive criterion—e.g., in 2014, as a response to the disasters, pumps failed, etc. The reason for such a situation can be understood through the fact that there is a lack of interest in the evaluation process from funders who invest money in improving local capacities [76].

Following the examples and practices of the EU, since 2015 Serbia has been attempting to reform the domain of local self-government to better conform to the principles of good governance. Good governance refers to governance that is responsible, open, user-oriented, inclusive, and sensitive to the needs of the local community [77,78,79]. All of the above speaks in favor of the fact that the effective implementation of the concept of DRM policy in cities in Serbia requires the continuous strategic, tactical, and operational transformation of public administration and public management to strengthen local governments’ capacity for disaster prevention, preparation, response, and recovery. All these factors confirm the general hypothesis of this research. It is necessary to take into account the results of the research Kusumasari, Alam, and Siddiqui [80], who found that it was necessary to meet the capability requirements across all different stages of disaster management (mitigation stage: evaluation, monitoring, and dissemination; preparedness stage: planning, exercise, and training; response stage: need assessment, information exchange, and logistical expertise; recovery stage, expertise in damage assessment and debris removal, disaster assistance skills, etc.

The increased risk of disasters, combined with the increasing security vulnerabilities of people and their property [81], creates the need for society in Serbia to be responsible in order to improve resilience. In order to accomplish this, a thorough examination of people’s needs and the options for upgrading the existing DRM system is required. The limitations of the existing normative framework in the field of legal regulation in disaster risk management, as well as the inefficient and untimely implementation and realization of legal solutions, suggest the necessity of the realization of more serious scientific research projects (e.g., DAREnet, DISARIMES) with the aim of designing concrete and applicable practical solutions. Furthermore, there is a growing need to shift the system’s operation from a reactive to a proactive strategy that implements actions and activities to decrease the risk of catastrophes.

Considering the rich treasury of results obtained, it can be said that the current situation requires the improvement of existing systems for disaster risk management by undertaking a wider range of activities and measures rather than simply traditional structural (the design, construction, maintenance, and renovation of physical structures) and nonstructural (urban planning and regulations, population preparation, improved forecasting, etc.) solutions. We need innovative solutions which include the use of modern innovative technical and managerial techniques (Table 8).

The limitations of our study include (1) its broad research framework; (2) the fact that perception of disaster preparedness largely depends on the place of the individual in the system; (3) the fact that no additional research has been conducted on how local governments react to disasters in relation to political, institutional, time, information, and disaster complexity dimensions; (4) the lack of research examining the attitudes of citizens to the existing capacities of local communities for disasters.

## 5. Conclusions

The normative and functional incompleteness and partial nonestablishment of the DRM system at the central level has resulted in the fact that the system has not been established at the local level to a great extent either. Disaster risk management at the local level is carried out in a uniform manner: all local government units have the same type and scope of competencies. Generally, no differences were perceived between towns and municipalities with respect to the degree of establishment of competencies, whether normatively, institutionally, functionally, operationally, or financially, except in individual cases in favor of municipalities, which should be the subject of separate research.

Strategic documents in the domain of community safety are not mandatory and do not exist in large numbers, whereas mandatory ones (crucial to the prevention of disasters), such as risk assessments and protection and rescue schemes, have been adopted to an alarmingly small degree. The competencies of towns in the operational sense, such as the establishment of headquarters and the adoption of accompanying documents, have only been partially fulfilled. Obligations that reflect the functional establishment of competencies that require full political, organizational, and financial commitment and the incorporation of the security concept into the organizational system have not been established. This refers primarily to the operation and equipment of all-purpose civil protection units, emergency population warning systems, education, etc. Supervision over the exercise of competencies in the domain of disasters in local self-government units is not visible in the system. Additionally, liability does not exist in practice, and neither do the procedures and mechanisms for determining the fulfillment of the legally prescribed competencies of local self-government units. Individual, legal, and criminal liabilities have not been made clear, with the assumed liability being primarily political in nature.

Cooperation among the different levels of government was assessed as good, as is coordination. Cooperation with neighboring municipalities during disaster events was deemed to be poor (less than half of the local self-government units participating in the survey stated that they cooperate with other municipalities), while preventive intermunicipal cooperation was almost symbolic in nature. There was no cross-border cooperation, and there is plenty of room for improvement in this area. Knowledge about the existence of international funds for project financing was insufficient. Regarding project financing, town representatives were more informed than municipal ones, but interest in the proposal and implementation of projects was generally low. Employees in local self-government units were not additionally motivated to develop projects, as they generally will be given more money for extra work. The projects were developed mainly by the local economic development offices, which serve as developmental organizational units, and were implemented by various sectors of local self-governments within the project-related area.

Additional motivation is needed to encourage employees in local self-government units to develop and implement projects in the field of DRM. Cooperation and communication among participants at the local level were reduced to formalized meetings, with no substantial insight into the capacities of the subjects and their vulnerability assessment schemes and a lack of developed informal contacts among people prior to a disaster. Resource databases for DRM do not exist. Based on conversations with subjects at the central level, it is assumed that each institution and protection and rescue formation at the local level is acquainted with the contents of its databases of available resources (which are not necessarily digital). The recommendation is that attention should be called to the establishment of standardized, available, and electronic resource databases for local DRM.

Cities have formally improved their competencies in areas that do not require large investments and resources, such as holding staff meetings, appointing legal entities, and appointing civil protection commissioners and deputies. When it comes to forming expert-operational teams and standing commissions for damage assessment, they have remained at the same level or progressed, but not significantly. In areas that require serious political, professional, and public commitment and significant allocations, such as formation, the training and equipping of general-purpose civil protection units, situation centers, acoustic studies, the creation of an alert system, personal and collective protection, and simulation exercises, the situation is generally unsatisfactory. The competencies of local self-government in the operational and tactical sense are not sufficient. The strategic risk assessment plans provided by law have not been sufficiently implemented or adopted in a timely manner in cities, so the style of crisis management is mostly reactive. Organizational constraints relating to disaster risk management include a lack of staff, insufficient staff education, and financial and technical constraints. Failure to recognize the importance of prevention by policymakers is one of the most important organizational constraints. Additionally, organizational memory, as an integral part of the cognitive dimension of the learning of local self-government units in disasters and its explicit form (such as rulebooks, manuals, instructions, public policy strategic documents, databases) has not been sufficiently established.

On the other hand, cooperation and communication between actors at the local level have been reduced to formalized meetings, without substantial insight being gained into the capacities of actors, vulnerability assessments being carried out, or developed informal contacts being created between people before disasters occur. Resource databases for disaster risk management are not sufficient. Based on conversations with actors at the central level, it is assumed that each institution and player in protection and rescue at the local level knows what it must do and has its own database of available resources (not necessarily electronic).

Through this research, we aim to enable both decision makers and citizens to competently and effectively respond to challenges of existing DRM policy in accordance with the principles of prevention, proactive action, coordination, cooperation, partnership, and responsibility, as well as to suggest new research perspectives.

## Figures and Tables

**Table 1:** Overview of the basic characteristics of local self-governments (*n* = 105).

Variable	Category	(*f*)	%
Type of local self-government	Municipality	83	79.05
	Town	25	20.95
Allocates budget funds for DRM	Yes	78	74.29
	No	27	25.71
Legislation	Fully and appropriately regulates	31	29.52
	Incompletely or inadequately regulates	74	70.48
Disaster risk assessment	Made	37	34.91
	Unmade	69	65.09
Disaster headquarters	Established	102	97.14
	Not established	3	2.86
Cooperation with relevant subjects	Exists	87	82.86
	Does not exist	18	17.14
Observed needs of vulnerable groups	Observed	49	46.67
	Not observed	56	53.33
Enhance communal companies in disaster	Yes	26	24.76
	No	79	75.24
TOTAL		105	100

**Table 2:** Results of a multivariate regression analysis of the capacity development of local self-governments for DRR.

Predictor Variable	Preparedness of Local Self-Government			Obligations of Local Self-Government			Received Support		
	*B*	*SE*	*β*	*B*	*SE*	*β*	*B*	*SE*	*β*
Budget funds	0.136	0.078	0.116	0.701	0.144	0.445 **	0.395	0.351	0.193
Cooperation	0.042	0.073	0.038	0.000	0.135	0.000	−0.196	0.343	−0.110
Disaster risk assessment	0.024	0.072	0.023	0.032	0.132	0.023	0.137	0.305	0.078
Protection and rescue	0.025	0.105	0.016	0.209	0.194	0.102	−0.312	0.482	−0.122
Assessment of legislation	0.167	0.074	0.150 *	−0.046	0.137	−0.031	0.238	0.374	0.121
Headquarters preparedness	0.107	0.070	0.105	−0.092	0.129	−0.068	0.381	0.401	0.217

* *p* ≤ 0.05; ** *p* ≤ 0.01; *B*: unstandardized (*B*) coefficients; *SE*: std. error; *β*: standardized (*β*) coefficients. Note: having allocated budget funds, having a great extent of cooperation, having finished a disaster risk assessment, having finished a protection and rescue plan, having full and appropriate legislation, and having a fully prepared headquarters have been given the code of 1; a value of 0 has been assigned otherwise.

**Table 3:** Pearson’s correlation results for the relationship between the capacity development of local self-governments for DRM and predictor variables.

		Preparedness of Local Self-Government		Obligations of Local Self-Government		Received Support		Headquarters Preparedness
Variables	Sig.	*r*	Sig.	*r*	Sig.	*r*	Sig.	*r*
Budget funds	0.019 *	0.232	0.000 **	0.444	0.114	−0.272	0.152	0.144
Disaster risk assessment	0.139	−0.152	0.549	−0.062	0.388	−0.155	0.606	0.054
Protection and rescue	0.053	−0.200	0.303	−0.107	0.352	0.173	0.657	0.047
Rules of procedures	0.336	−0.098	0.738	0.034	0.985	−0.003	0.601	0.010
Annual work plan	0.041 *	−0.205	0.282	−0.109	0.327	0.173	0.229	0.123
Civil protection	0.276	−0.110	0.941	−0.008	0.508	0.114	0.111	0.162
Duties of headquarters	0.171	−0.140	0.557	0.060	0.122	0.270	0.026 *	0.222
Extent of cooperation	0.061	−0.185	0.751	−0.032	0.257	−0.194	0.395	0.086

* *p* = 0.05; ** *p* ≤ 0.01.

**Table 4:** Independent samples *t*-test results comparing towns and municipalities of Serbia regarding various dimensions.

		N	M	SD	*t*	*df*	*p*
General preparedness	Municipalities	83	2.95	0.516	−1.182	101	0.782
	Towns	20	3.10	0.447			
Awareness on the law on disaster risk reduction	Municipalities	83	3.25	0.696	−4.053	101	0.000 **
	Towns	20	3.90	0.308			
Allocated funds from the local budget	Municipalities	32	0.897	1.001	1.931	41	0.060
	Towns	11	0.295	0.324			
Communication assessment results	Municipalities	77	3.19	0.514	−0.829	95	0.409
	Towns	20	3.30	0.470			

** *p* ≤ 0.01.

**Table 5:** Review of appropriate acts carried out.

Document Title	%
Disaster management headquarters were established	99.1
Rules of procedure were issued	99.1
Annual work report was completed	83.3
Annual work plan was completed	88.9
Decision for organizing and operating general-purpose civil protection was made	95.0
Decision for establishing general-purpose civil protection units was made	75.0
Civil protection commissioner was appointed	94.7
Duties were assigned to members of disaster management headquarters	88.2
Teams were set up for disaster risk assessment and protection and rescue plans were drawn up	72.2
Threat assessment document was issued	36.8
Protection and rescue schemes were issued	16.7
An operational flood response scheme for second-order streams was adopted	90.0

**Table 6:** Chi-square test results relating to disaster management units on the degree of awareness of local self-government representatives.

Variable	Sig. (2-Tailed)	*df*	*X* ^2^
Decision on formation of the headquarters passed	0.50 *	2	5.99
Rules of procedure adopted	0.96	2	0.61
Annual work report adopted	0.191	2	3.31
Annual work plan adopted	0.102	2	4.56
Decision on formation and operation of civil protection made	0.500	2	0.77
Decision on setting up a civil protection unit passed	0.134	2	4.02
Conclusion made on the appointment of the civil protection commissioner	0.672	2	0.79
Duties assigned to members of disaster management headquarters	0.028 *	2	7.14
Risk assessment team formed	3.38	2	2.17
Risk assessment adopted	0.277	2	2.56
Protection and rescue scheme adopted	0.066	2	5.43
Flood defense scheme adopted	0.770	2	0.523
Legislation in power in the domain of disaster management assessed	0.022 *	2	7.63
Budget financing	0.050 *	2	5.99
Having insight into international funds intended for improving readiness	0.272	2	2.60
Steps taken to improve quality of Town planning schemes	0.050 *	2	6.01
Support in developing schemes	0.118	2	4.27
Having insight into the competencies of disaster headquarters	0.530	2	1.27
Disaster management team established	0.050 *	2	5.91
Assessment of readiness of disaster management headquarters	0.050 *	4	9.13
Assessment of established communication	0.001 **	2	9.59
Assessment of cooperation with other municipalities	0.282	4	5.05
Cooperation with other municipalities in the domain of disaster prevention	0.647	4	2.48
Assessment of cooperation with cross-border municipalities	0.551	2	1.19
Assessment of the need for cross-border cooperation	0.668	6	4.06
Cooperation with governmental institutions	0.043 *	2	6.28
Involving citizens in disaster prevention framework	0.075	2	5.18

* *p* ≤ 0.05; ** *p* ≤ 0.01.

**Table 7:** Attitudes of heads (mayor) of disaster sectors in local self-government units.

Mayor	Legal Solution	City Administration	Legally Regulated Jurisdiction	Implemented Jurisdiction	Strategic Plans	Training and Education	Citizen Engagement	Civil Society Organizations	Total
Beograd	4	4	2	4	4	2	2	4	3.3
Čačak	5	5	4	5	4	4	4	5	4.5
Kikinda	5	4	4	5	4	3	4	5	4.3
Kragujevac	3	4	4	1	3	4	4	5	3.5
Kraljevo	5	4	4	4	4	4	4	5	4.3
Kruševac	5	5	2	4	3	4	2	5	3.8
Leskovac	5	4	3	3	4	3	3	4	3.6
Loznica	5	4	4	4	4	3	4	4	4.0
Niš	5	4	4	4	4	4	4	5	4.3
Novi Pazar	5	4	4	4	3	3	4	5	4.0
Pančevo	5	4	4	5	4	5	3	5	4.4
Sombor	5	5	5	4	4	2	5	5	4.4
Sremska Mitrovica	5	5	4	5	4	4	4	5	4.5
Zrenjanin	5	4	4	4	4	5	4	5	4.4
Vranje	5	4	4	4	3	4	4	5	4.1
Užice	5	4	4	4	3	3	3	5	3.9
Šabac	5	4	5	5	5	5	5	5	4.9
Total	4.8	4.2	3.8	4.1	3.8	3.6	3.7	4.8	/

**Table 8:** Recommendations for improving the situation in the field of disaster risk management in Serbia.

	Specific Actions
A. Improve, strengthen, and enhance resilience through:	1. Commitment to disaster risk reduction policy by key actors (authorities, local governments, legal entities).
	2. Effective coordination and operational cooperation between all entities and protection and rescue forces.
	3. Conditions for the consistent and efficient application of regulations and the organization of preventive measures.
	4. Infrastructure, equipment, means of protection and rescue, specialized cadasters and risk maps.
	5. Training and qualification of specialized staff as well as a culture of prevention.
	6. Risk assessment methodology and the development of protection and rescue plans, as well as hazardous waste management methodology.
	7. Financing of the protection and rescue system.
	8. International scientific cooperation in the field of disaster prevention.
	9. Disaster protection and rescue plans to be adopted for all local governments following specific scenarios of security threats.
	10. Supervision over the exercise of competencies of local self-government units in the field of disasters.
	11. Joint exercises and training, competitions, and sports games where members of various public services can practice.
	12. Involvement of citizens in decision-making processes (disaster risk management) at the local level.
	13. Establishment of standardized, accessible, and electronic databases of resources for disaster management at the local level.
	14. System of learning from previous disasters based on quality and objective analyses.
B. Build, develop, and implement disaster risk reduction strategies through:	1. The unique phone number 112 that citizens could call in emergencies.
	2. Operational response procedures in various disasters that will allow the coordinated, rapid, and efficient intervention of all competent services.
	3. Improved communication systems to inform and alert citizens.
	4. The use of a variety of communication methods to improve the dissemination of hazard-related information and necessary level of preparedness.
	5. Procedures and mechanisms for determining the fulfillment of the legally prescribed competencies of local self-government.
	6. Innovative technical solutions for monitoring various dangers, informing and alerting citizens, and protection and rescue (structural and nonstructural solutions).
	7. Including disaster education in children’s school curricula.
	8. Dialogue and cooperation between scientific and relevant subjects.
	9. Developing scientific capacities and investing in innovation and technological development.
	10. Local and national citizen awareness campaigns.

## Supplementary Materials

The following are available online at https://www.mdpi.com/article/10.3390/ijerph181910406/s1. Figure S1. Study areas location. Numbers refer to the ID of the municipality. Table S1. The ID of the municipality and belonging county in Serbia. Questionnaire S1. The Questionnaire Intended for Representatives of Local Self-Government Units in Towns Relative to Disaster Management Activities

## Appendix A

**Table A1:** Attitudes of heads (mayors) of disaster sectors in local self-government units.

Attitudes	1 = Strongly Disagree	2	3	4	5 = Strongly Agree
The legal solution according to which the mayor is the commander of the disaster headquarters is good and should not be changed.					
The city administration across all sectors is fully prepared to respond to disasters.					
The competencies of the local self-government in disaster management in Serbia are fully and sufficiently precisely regulated by laws.					
The city’s disaster management processes are sufficient (it has formed operational expert teams, appointed commissioners of civil protection and their deputies, formed and equipped and trained civil protection units, created a situation center, created a means of alert, etc.).					
Strategic risk assessment plans provided by law are adopted and implemented in cities sufficiently and in a timely manner and the style of disaster management is mostly proactive.					
Actors in the local self-government (public administration, public services and policy makers, citizens) are sufficiently trained and educated on disaster management.					
Activities and voluntary engagement of citizens in emergency situations are appropriately regulated by laws and appropriate standard procedures.					
Civil society organizations and citizens’ associations, volunteers are very important in disasters.					

## Appendix B

**Table A2:** An overview of socioeconomic and health indicators (2017) [82].

No.	Towns	Estimated No. Population 2015	Budget for 2015 in RSD	Per km^2^/Percentage Share of the Total Territory of RS	Density of Population	Number of Settlements	Number of Unemployed Registered with the NES in 2015	No. of Beneficiaries of Social Necessary Assistance	Life Expectancy of Newborns 2013–2015
1	Beograd	1,679,895	84,083,697,000	3234/3.65%	519	17 municipalities	105,724		Male 73.75
						157 settlements	6.3%		Female 78.57
2	Valjevo	87,944	2,338,643,000	905/1.02%	97	78 settlements	7800	790	Male 72.91
							8.87%		Female 78.11
3	Vranje	81,986	2,228,913,000	860/0.97%	95	2 municipalities	8801	1271	Male 72.96
						105 settlements	10.7%		Female 78.03
4	Zaječar	56,714	1,378,590,000	1069/1.21%	53	42 settlements	7020	1368	Male 71.13
							12.38%		Female 78.43
5	Zrenjanin	119,710	3,831,148,000	1327/1.49%	90	22 settlements	7797	2490	Male 70.66
							6.51%		Female 77.22
6	Jagodina	70,772	2,196,834,000	470/0.53%	151	53 settlements	10,432	1320	Male 72.47
							14.74%		Female 77.69
7	Kikinda	56,760	2,086,064,000	783/0.88%	72	10 settlements	5554	1640	Male 70.76
							9.78%		Female 76.59
8	Kragujevac	178,610	5,851,240,000	835/0.94%	214	57 settlements	22,121	1876	Male 73.94
							12.38%		Female 77.92
9	Kraljevo	121,766	2,763,582,000	1530/1.73%	80	92 settlements	12,844	1390	Male 73.70
							10.55%		Female 78.42
10	Kruševac	124,795	2,324,717,000	854/0.96%	146	101 settlements	15,704	4093	Male 73.40
							12.58%		Female 78.16
11	Leskovac	139,291	3,085,084,000	1025/1.16%	136	144 settlements	19,151	2090	Male 72.34
							13.75%		Female 76.73
12	Loznica	76,958	1,679,803,000	612/0.69%	126	54 settlements	11,936	1831	Male 72.60
							15.51%		Female 77.70
13	Novi Pazar	103,892	2,114,736,000	742/0.84%	140	99 settlements	18,437	2750	Male 73.09
							17.75%		Female 77.35
14	Novi Sad	350,930	15,841,641,000	699/0.79%	502	2 municipalities	26,406	5034	Male 73.36
						16 settlements	7.5%		Female 78.62
15	Niš	257,883	8,143,216,000	596/0.67%	433	5 municipalities	32,111	12,539	Male 74.10
						71 settlements	12.45%		Female 78.25
16	Pančevo	121,482	4,255,613,000	759/0.85%	161	10 settlements	10,590	995	Male 72.36
							8.72%		Female 77.57
17	Pirot	55,885	1,537,859,000	1232/1.39%	45	72 settlements	7206	4000	Male 73.40
							12.89%		Female 79.29
18	Smederevo	105,774	2,824,240,000	484/0.55%	219	28 settlements	8763	3325	Male 72.30
							8.28%		Female 77.58
19	Sombor	82,389	2,074,954,000	1216/1.37%	68	16 settlements	7130	3982	Male 71.06
							8.65%		Female 77.34
20	Sremska Mitrovica	77,667	2,156,562,000	762/0.86%	102	26 settlements	6564	1907	Male 71.95
							8.45%		Female 77.09
21	Subotica	139,011	4,298,677,000	1007/1.14%	138	19 settlements	8879	4213	Male 70.81
							6.39%		Female 76.52
22	Užice	75,805	2,329,991,000	667/0.75%	114	41 settlements	4873	463	Male 73.95
							6.43%		Female 78.73
23	Čačak	112,558	2,581,053,000	636/0.72%	176	58 settlements	10,470	580	Male 73.38
							9.30%		Female 78.42
24	Šabac	113,113	2,784,315,000	797/0.90%	142	52 settlements	11,361	1916	Male 71.47
							10.04%		Female 76.81
25	Srbija	7,095,383	245,365,641,000	88,499/100%	91	6158 settlements	703,020	106,439	Male 72.62
							9.91%	3.5%	Female 77.67
