# Supplementary material for: Capacity Development of Local Self-Governments for Disaster Risk Management
Source: Int J Environ Res Public Health. 2021 Oct 2;18(19):10406. doi: 10.3390/ijerph181910406 (PMC8508225; doi:10.3390/ijerph181910406)
Supplement: Supplementary file 1 [file ijerph-18-10406-s001.zip › ijerph-1338368-supplementary.pdf]

International Journal of
Environmental Research and Public Health

MDPI

Supplementary material

Capacity Development of Local Self-governments in the Field of Disaster Risk Management

Vladimir M. Cvetković [1,2,*], Jasmina Tanasić [3], Adem Ocal [4], Želimir Kešetović [2], Neda Nikolić [5] and Aleksandar Dragašević [5]

[1] Faculty of Security Studies, University of Belgrade, Gospodara Vučića 50, 11040 Belgrade, Serbia; vmc@fb.bg.ac.rs; zelimir.kesetovic@fb.bg.ac.rs (Ž.K.)
[2] Scientific-Professional Society for Disaster Risk Management, Dimitrija Tucovića 121, 11040 Belgrade, Serbia; upravljanje.rizicima.vs@gmail.com
[3] Standing Conference of Towns and Municipalities, 11103 Belgrade, Serbia; jasmina.tanasic@skgo.org
[4] Independent Researcher, Ankara 06500, Turkey; ocadem@gmail.com
[5] Faculty of Technical Sciences, University of Kragujevac, 32102 Čačak, Serbia; neda.nikolic@ftn.kg.ac.rs (N.N.); aleksandar.dragasevic@ftn.kg.ac.rs (A.D.)

* Correspondence: vmc@fb.bg.ac.rs

Citation: Cvetković, V.M.; Tanasić, J.; Ocal, A.; Kešetović, Ž.; Nikolić, N. Capacity Development of Local Self-Governments for Disaster Risk Management. **2021**, *18*, 10406. https://doi.org/10.3390/ijerph181910406

Academic Editor: Paul B. Tchounwou

Received: 28 July 2021
Accepted: 16 September 2021
Published: 2 October 2021

Publisher's Note: MDPI stays neutral with regard to jurisdictional claims in published maps and institutional affiliations.

Copyright: © 2021 by the authors. Submitted for possible open access publication under the terms and conditions of the Creative Commons Attribution (CC BY) license (http://creativecommons.org/licenses/by/4.0/).

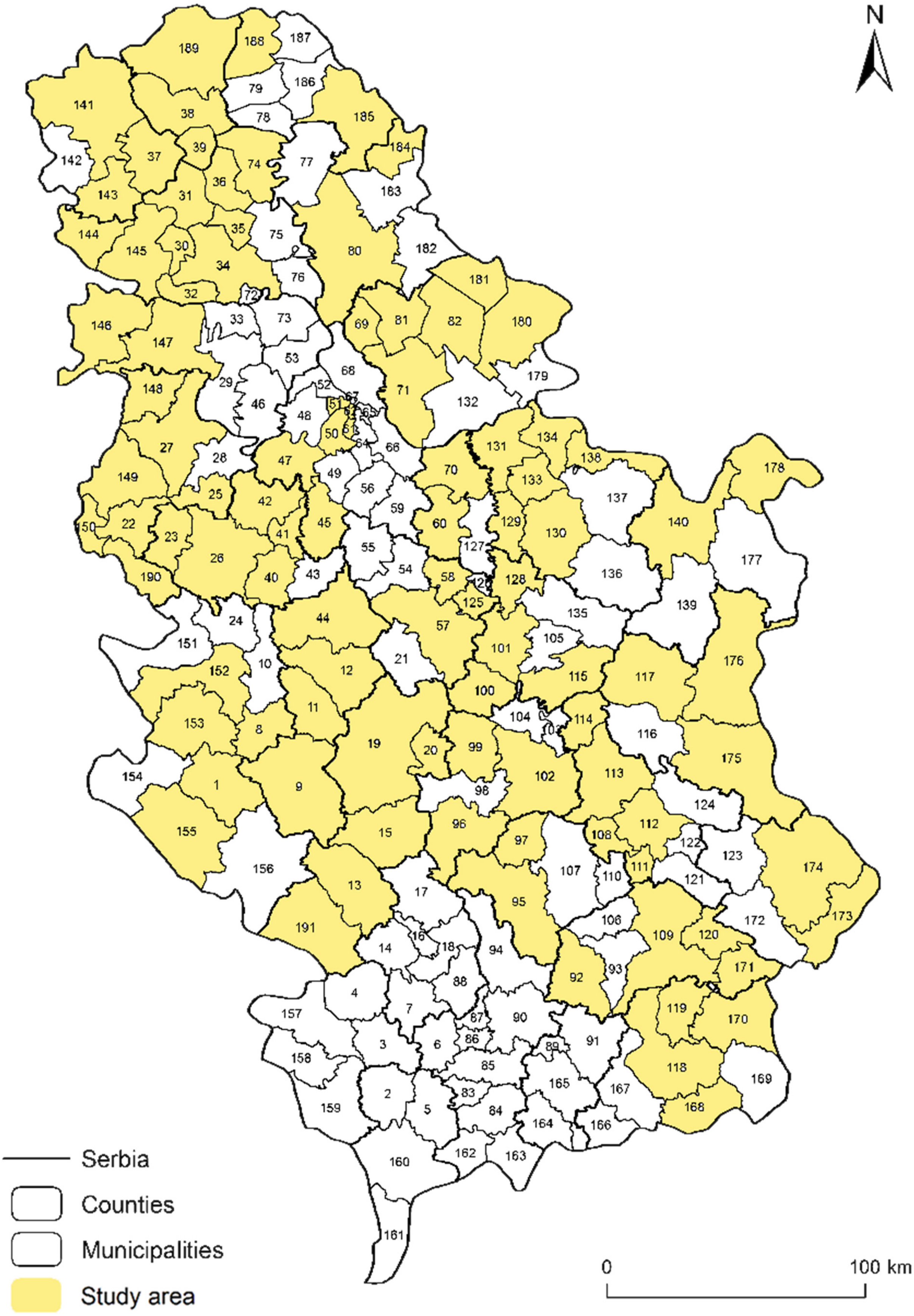

Figure S1. Study areas location. Numbers refer to the ID of the municipality.

Table S1. The ID of the municipality and belonging county in Serbia.

ID	Municipality	County	ID	Municipality	County
1	Nova Varoš	Zlatibor	96	Brus	Rasina
2	Orahovac	Prizren	97	Blace	Toplica
3	Klina	Peč	98	Aleksandrovac	Rasina
4	Istok	Peč	99	Trstenik	Rasina
5	Suva Reka	Prizren	100	Rekovac	Pomoravlje
6	Glogovac	Kosovo	101	Jagodina	Pomoravlje
7	Srbica	Kosovo-Mitrovica	102	Kruševac	Rasina
8	Arilje	Zlatibor	103	Ćicevac	Rasina
9	Ivanjica	Moravica	104	Varvarin	Rasina
10	Požega	Zlatibor	105	Ćuprija	Pomoravlje
11	Lučani	Moravica	106	Bojnik	Jablanica
12	Čacak	Moravica	107	Prokuplje	Toplica
13	Novi Pazar	Raška	108	Merošina	Nivaša
14	Zubin Potok	Kosovo-Mitrovica	109	Leskovac	Jablanica
15	Raška	Raška	110	Žitoradja	Toplica
16	Zvečan	Kosovo-Mitrovica	111	Doljevac	Nivaša
17	Leposavić	Kosovo-Mitrovica	112	Niš	Nivaša
18	Kosovska Mitrovica	Kosovo-Mitrovica	**113**	**Aleksinac**	Nivaša
19	Kraljevo	Raška	114	Ražanj	Nivaša
20	Vrnjačka Banja	Raška	115	Paraćin	Pomoravlje
21	Knić	Šumadija	116	Sokobanja	Zalečar
22	Krupanj	Mačva	117	Boljevac	Zalečar
23	Osecina	Kolumbara	118	Vranje	Pčinja
24	Kosjerić	Zlatibor	119	Vladičin Han	Pčinja
25	Koceljeva	Mačva	120	Vlasotince	Jablanica
26	Valjevo	Kolumbara	121	Gadzin Han	Nivaša
27	Šabac	Mačva	122	Niška Banja	Nivaša
28	Vladimirci	Mačva	123	Bela Palanka	Pirot
29	Ruma	Srem	124	Svrljig	Nivaša
30	Bački Petrovac	South Bačka	125	Batočina	Šumadija
31	Vrbas	South Bačka	126	Lapovo	Šumadija
32	Beočin	South Bačka	127	Velika Plana	Podunavlje
33	Irig	Srem	128	Svilajnac	Pomoravlje
34	Novi Sad	South Bačka	129	Žabari	Braničevo
35	Temerin	South Bačka	130	Petrovac	Braničevo
36	Srbobran	South Bačka	131	Pozarevac	Braničevo
37	Kula	West Bačka	132	Kovin	South Banat
38	Bačka Topola	North Bačka	133	Malo Crnice	Braničevo
39	Mali Iđoš	North Bačka	134	Veliko Gradiste	Braničevo
40	Mionica	Kolumbara	135	Despotovac	Pomoravlje
41	Lajkovac	Kolumbara	136	Zagubica	Braničevo
42	Ub	Kolumbara	137	Kučevo	Braničevo
43	Ljig	Kolumbara	138	Golubac	Braničevo
44	Gornji Milanovac	Moravica	139	Bor	Bor
45	Lazarevac	City of Belgrade	140	Majdanpek	Bor
46	Pecinci	Srem	141	Sombor	West Bačka
47	Obrenovac	City of Belgrade	142	Apatin	West Bačka
48	Surčin	City of Belgrade	143	Odzaci	West Bačka
49	Barajevo	City of Belgrade	144	Bač	South Bačka

50	Čukarica	City of Belgrade	145	Bačka Palanka	South Bačka
51	Novi Beograd	City of Belgrade	146	Šid	Srem
52	Zemun	City of Belgrade	147	Sremska Mitrovica	Srem
53	Stara Pazova	Srem	148	Bogatic	Mačva
54	Topola	Šumadija	149	Loznica	Mačva
55	Arandjelovac	Šumadija	150	Mali Zvornik	Mačva
56	Sopot	City of Belgrade	151	Bajina Bašta	Zlatibor
57	Kragujevac	Šumadija	152	Užice	Zlatibor
58	Raca	Šumadija	153	Čajetina	Zlatibor
59	Mladenovac	City of Belgrade	154	Priboj	Zlatibor
60	Smederevska Palanka	Podunavlje	155	Prijepolje	Zlatibor
61	Rakovica	City of Belgrade	156	Sjenica	Zlatibor
62	Savski Venac	City of Belgrade	157	Peć	Peč
63	Vračar	City of Belgrade	158	Dečani	Peč
64	Voždovac	City of Belgrade	159	Djakovica	Peč
65	Zvezdara	City of Belgrade	160	Prizren	Prizren
66	Grocka	City of Belgrade	161	Gora	Prizren
67	Stari Grad	City of Belgrade	162	Strpce	Kosovo
68	Palilula	City of Belgrade	163	Kačanik	Kosovo
69	Opovo	South Banat	164	Vitina	Kosovo-Pomoravlje
70	Smederevo	Podunavlje	165	Gnjilane	Kosovo-Pomoravlje
71	Pančevo	South Banat	166	Preševo	Pčinja
72	Sremski Karlovci	South Bačka	167	Bujanovac	Pčinja
73	Indjija	Srem	168	Trgovšte	Pčinja
74	Bečej	South Bačka	169	Bosilegrad	Pčinja
75	Abalj	South Bačka	170	Surdulica	Pčinja
76	Titel	South Bačka	171	Crna Trava	Jablanica
77	Novi Bečej	Central Banat	172	Babušnica	Pirot
78	Ada	North Banat	173	Dimitorvgrad	Pirot
79	Senta	North Banat	174	Pirot	Pirot
80	Zrenjanin	Central Banat	175	Knjaževac	Zaječar
81	Kovačica	South Banat	176	Zaječar	Zaječar
82	Alibunar	South Banat	177	Negotin	Bor
83	Stimlje	Kosovo	178	Kladovo	Bor
84	Urosevac	Kosovo	179	Bela Crkva	South Banat
85	Lipljan	Kosovo	180	Vršac	South Banat
86	Kosovo Polje	Kosovo	181	Plandište	South Banat
87	Obilić	Kosovo	182	Sečanj	Central Banat
88	Vučitrn	Kosovo-Mitrovica	183	ÄitiÜte	Central Banat
89	Novo Brdo	Kosovo-Pomoravlje	184	Nova Crnja	Central Banat
90	Pristina	Kosovo	185	Kikinda	North Banat
91	Kosovska Kamenica	Kosovo-Pomoravlje	186	Čoka	North Banat
92	Medveđa	Jablanica	187	Novi Kneževac	North Banat
93	Lebane	Jablanica	188	Kanjiža	North Banat
94	Podujevo	Kosovo	189	Subotica	North Bačka
95	Kuršumlija	Toplica	190	Ljubovija	Mačva
			191	Tutin	Raška

Questionnaire S1. **The Questionnaire Intended for Representatives of Local Self-Government Units in Towns Relative to Disaster Management Activities**

Degree of Preparedness

1. In your opinion, how prepared is your local self-government unit for disaster risk management? Please answer by circling one of the offered options:

    It is completely unprepared

    It is unprepared
    It is prepared
    It is completely prepared

2. In your opinion, what are the main problems and obstacles to enhancing the capacities of your local self-government units in disaster risk management? Please list up to five issues in order of importance (starting from the one you consider most important).

    1. ______________________________
    2. ______________________________
    3. ______________________________

Legal Framework

3. How informed are you about the obligations of your local self-government that arise from the law on disasters? Please answer by circling one of the offered options.

    1. I am not informed at all
    2. I am insufficiently informed
    3. I am pretty well informed
    4. I am fully informed

4. Please answer whether or not the following acts have been passed in your local self-government and which:

Document title:		
The act was passed	1—Yes	2—No
4.1. Decision on the formation of disaster risk management headquarters	1—Yes	2—No
4.2. Rules of Procedure of the disaster management headquarters	1—Yes	2—No
4.3. Annual work report	1—Yes	2—No
4.4. Annual work plan	1—Yes	2—No
4.5. Decision on the organization and operation of all-purpose civil protection	1—Yes	2—No
4.6. Decision on the establishment of all-purpose civil protection units	1—Yes	2—No
4.7. Conclusion on the appointment of the civil protection commissioner	1—Yes	2—No
4.8. Assignment of duties to the headquarters members	1—Yes	2—No
4.9. Mayor's decision on the formation of a team for the development of the vulnerability assessment and protection and rescue schemes	1—Yes	2—No
4.10. Disaster risk assessment	1—Yes	2—No
4.11. Protection and rescue plan	1—Yes	2—No
4.12. Flood defense scheme (second-order waterstreams)	1—Yes	2—No

5. If the following documents were adopted in your local self-government unit, please indicate whether the consent of the Ministry of Interior Sector has been obtained. 1—Yes 2—No

Document Title:

5.1. Vulnerability assessment scheme	1—Yes	2—No
5.2. Protection and rescue scheme	1—Yes	2—No

6. How do you assess the legislation in power (please choose one of the answers and explain):

    6.1. Fully and appropriately regulates the domain of disasters (please, provide and explanation)
    6.2. In some respects it is incomplete/inadequate (please, provide an explanation)

7. Please state the forms of assistance you need in order to improve/prepare local programs appropriately (multiple answers are possible; you may propose additional forms of assistance).

    7.1. Legislative framework (decisions, rules of procedure, instructions, etc.)
    7.2. Institutional framework (formation of a service, department or a specific organization, civil protection, etc.)
    7.3. Educational framework (new qualified staff, courses, licenses, trainings, workshops, round tables, public hearings, etc.)

7.4. Functional framework (lack of understanding on part of the authorities, inability to adopt a legal act, inadequate qualifications or equipment, etc.)
7.5. Other forms/please write…

Financial Framework

8. Does your town/municipality allocate budget funds for disaster risk management and mitigation in the period after a disaster? 1—Yes 2—No
9. If YES, please indicate the amount and proportion of the funds allocated from the local budget in the last fiscal year: ……………………..………………….. RSD%.
10. Please indicate the purpose of the stated budget funds (circle all appropriate answers):

1. Procurement of funds for civil protection purposes
2. Financing the hail protection system
3. Maintenance of the emergency population warning system
4. Waterstreams and critical infrastructure management
5. Promotional materials cost
6. Projects development
7. Miscellaneous

11. Are you informed about the funds/international funds available to local self-government units for the improvement of systems for responses to emergencies and disaster prevention? 1—Yes 2—No
12. If so, did your local self-government apply for funding? 1—Yes 2—No
13. If yes, please state the funds applied for and the source.

1. ____
2. ____
3. ____

Vulnerability Assessment and Protection and Rescue Schemes

14. If the vulnerability assessment and protection and rescue schemes have been adopted, has your local self-government taken any steps to enhance the quality of town planning schemes or the response and operation schemes of communal companies during disasters?
1—Yes (go to next question) 2—No (go to question 22)
15. Please answer regarding the availability of disaster risk assessment and protection and rescue schemes in each of the communal companies?
16. In addition to the communal companies, do you consider any other institution/organization of your local self-government to be relevant to the response to disasters and their prevention?
1—Yes (please, specify) 2—No (go to question 18)
17. If yes, are there vulnerability assessment and protection and rescue schemes for the members listed?
1—Yes, in all of the stated 2—Yes, in some of the above 3—No
18. Have you received any support in designing your vulnerability assessment and protection and rescue schemes so far? *1—Yes 2—No*
19. If YES, please indicate what that support included (multiple answers are possible; you may also propose other forms of support).

1. Institutional framework (please, specify the institutions from which the support was obtained)
2. Educational framework (please, list the forms of training required for the preparation of the document (please, specify the training form, who it was given to and when)
3. Functional framework (specify support in terms of information obtained and its processing, the required program tools needed for designing the documents)
4. Miscellaneous

20. How would you rate the support received so far (please circle only one answer)?
1. Completely insufficient 2. Insufficient 3. Relatively sufficient 4. Sufficient

Disaster Response Headquarters

21. Are you acquainted with responsibilities of the disaster headquarters? *1—Yes 2—No*
22. Is there a disaster headquarters in your local self-government? *1—Yes 2—No*
23. If YES, do you think that the headquarters is adequately prepared to respond to an emergency situation? *1. Yes, fully 2. Yes, partially 3. No*
24. Has the headquarters or local authority established communication with all the relevant institutions in the territory of your municipality/town (town council, assembly, administration departments, communal companies, institutions, etc.) in terms of the harmonization of legal acts

relative to disasters (prevention measures and those during and after disasters, mitigating the consequences of a disaster) *1—Yes* *2—No*

25. If the answer to the previous question is YES, please describe the form of communication used.
26. In your opinion, what effects have been achieved?
    1. Negligible 2. Minimal 3. Relatively good 4. Very good

Cooperation and Communication

27. To what extent have you cooperated with neighboring municipalities so far during a disaster?
    1. To a great extent, significantly
    2. To some extent, negligibly
    3. There was no cooperation at all
28. If there was cooperation, please describe it.
29. To what extent have you cooperated so far with neighboring municipalities in the domain of emergency prevention and management?
    29.1. To a great extent, significantly
    29.2. To some extent, negligibly
    29.3. There was no cooperation at all
30. If there was cooperation, please describe the form of the cooperation (multiple answers are possible).
    30.1. Planning and development of joint projects intended for financing prevention and emergency management systems;
    30.2. Planning and development of joint projects intended to finance relief in disaster;
    30.3. Joint trainings and drills;
    30.4. Miscellaneous
31. Have you cooperated with cross-border municipalities in the domain of emergency prevention and management to date?*1—Yes* *2—No*
32. If there has been cooperation with cross-border municipalities, please describe it (multiple answers are possible).
    32.1. Planning and development of joint projects intended for financing prevention and emergency management systems;
    32.2. Planning and development of joint projects intended to finance disaster relief;
    32.3. Joint trainings and drills;
    32.4. Miscellaneous
33. If there has been no cooperation with cross-border municipalities, do you think there is a need for such cooperation (one answer)?
    33.1. Yes, I think it is necessary
    33.2. Yes, it would be good to have
    33.3. No, it is difficult to achieve
    33.4. No, it is not needed
34. If you consider the cross-border cooperation in the domain of emergency prevention and management necessary, please make a proposal for the cooperation, if any.
35. Have you cooperated with any of the governmental institutions/bodies in charge of emergency management and prevention and/or with protection and rescue formations?
    1—Yes *2—No*
36. If you have cooperated in this way, please describe it (the form of cooperation) (multiple answers are possible if appropriate; optionally, you may propose other forms of cooperation as well if you deem it necessary).
    36.1. Legislative framework (participation in proposing laws and bylaws, i.e.,: decisions, rules of procedure, instructions, assessment schemes, etc.
    36.2. Institutional framework (enhancing the capacity of the existent formations in terms of participation in commissions, professional/operational teams, working groups, etc.)
    36.3. Educational framework (trainings, workshops, forums, public hearings, etc.)
    36.4. Functional framework (establishing and proper operation of the protection and rescue system, joint planning, response to emergencies and the relief in the aftermath
    36.5. Miscellaneous
37. Which governmental institutions/bodies did you cooperate with? Please specify.

38. Have you cooperated at all with the district disaster headquarters so far?
    1—Yes *2—No*
39. If YES, please state the form the cooperation took (multiple answers are possible; if appropriate, add other forms of cooperation).
    39.1. Legislative framework (activities for drafting the assessment, plans, acts of the staff, regulations, instructions, etc.):
    39.2. Institutional framework (formation of civil protection units, working bodies, commissions, expert-operational teams, etc.):
    39.3. Educational framework (trainings, workshops, forums, public hearings, etc.):
    39.4. Functional framework (establishment and adequate functioning of SZIS, joint planning, response and mitigating aftermath consequences):
    39.5. Miscellaneous
40. If you have not cooperated with the district disaster headquarters, please state the reasons why the cooperation has not been established to date.
41. Did you include citizens or consult with the wider community regarding disaster prevention and emergency management? 1—Yes 2—No
42. If YES, please indicate what forms their participation took.
    42.1. Meetings with citizens and community centers
    42.2. Organizing forums
    42.3. Educational workshops
    42.4. Civil protection trainings
    42.5. Civil protection disaster drills
    42.6. The work on vulnerability assessment and protection and rescue schemes
    42.7. Activities during a disaster and mitigating aftermath consequences:
    - *(a) notifying population on an emergency and the emergency warning*
    - *(b) evacuation*
    - *(c) critical infrastructure works*
    - *(d) sanitation practices*
    - *(e) emergency care*
    - *(f) relief supplies*
    - *(g) recovery of buildings*
43. In your opinion, are the employees and the officials in the town/municipality administration suitably qualified in the field of prevention and emergency management? 1—Yes 2—No
44. If the answer is NO, please state the ways you think the knowledge of employees and officials in the administration of your town/municipality could be improved (multiple answers are possible/other ways can be proposed too).
    44.1. Legislative framework (e.g., drafting new and amending existing acts)
    44.2. Institutional framework (incorporation of new institutions in to the existing framework, establishing new services or institutions, etc.)
    44.3. Educational framework (trainings, workshops, forums, public debates, etc.)
    44.4. Functional framework (engaging into making decision, implementation, supervision, etc.)
    44.5. Miscellaneous
45. Do you think that the population of your town/municipality is sufficiently educated in the matter of the prevention and management of emergencies? 1—Yes 2—No
46. If the answer is NO, please list the ways you think the awareness of the local community could be increased (multiple answers are possible).
    46.1. Institutional framework (involving civil society organizations in the processes, establishing new services or institutions, etc.)
    46.2. Educational framework (workshops, forums, public hearings, etc.)
    46.3. Miscellaneous
47. In your opinion, what institution/organization/services, etc., should be given priority in your local self-government in raising awareness on dangers, prevention, and protection in emergencies? Please rank these actors by assigning number 1 to the option with the highest priority and number 6 to the one with the lowest priority.
    47.1. Political leadership of the local administration
    47.2. Employees in the local administration
    47.3. Management of public companies and institutions

47.4. Representatives of local media
47.5. School children and youth
47.6. Citizens

48. If your municipality/town/municipal government has prepared a risk assessment, protection and rescue schemes, or long-term improvement and development plan, etc., please circle who was involved in the drafting of these documents.

48.1. Commission for Gender Equality
48.2. Person in charge of gender equality issues
48.3. Civil society organizations
48.4. Women's associations

49. If your municipality/town/municipal government has not developed a risk assessment, protection and rescue schemes, or a long-term improvement and development plan, etc., in your opinion are there any local institutions/organizations/associations that could contribute to understanding the different needs and statuses of women and men in disasters? Please choose out of the offered options the ones you consider to be appropriate:

49.1. Commission for Gender Equality
49.2. Person in charge of gender equality issues
49.3. Civil society organizations
49.4. Women's associations

50. To what extent are the needs of vulnerable groups (persons with disabilities) observed within the protection and rescue schemes and other aspects of emergency prevention and management (persons with special needs, persons with disabilities, Roma, etc.)?

50.1. Yes, their needs are fully observed
50.2. Yes, their needs are partially observed (specify where and how)
50.3. No
